# Genome-wide association studies and Mendelian randomization analyses provide insights into the causes of early-onset colorectal cancer

**DOI:** 10.1016/j.annonc.2024.02.008

**Published:** 2024-06

**Authors:** R.S. Laskar, C. Qu, J.R. Huyghe, T. Harrison, R.B. Hayes, Y. Cao, P.T. Campbell, R. Steinfelder, F.R. Talukdar, H. Brenner, S. Ogino, S. Brendt, D.T. Bishop, D.D. Buchanan, A.T. Chan, M. Cotterchio, S.B. Gruber, A. Gsur, B. van Guelpen, M.A. Jenkins, T.O. Keku, B.M. Lynch, L. Le Marchand, R.M. Martin, K. McCarthy, V. Moreno, R. Pearlman, M. Song, K.K. Tsilidis, P. Vodička, M.O. Woods, K. Wu, L. Hsu, M.J. Gunter, U. Peters, N. Murphy, Colorectal Transdisciplinary (CORECT) Study, the Colon Cancer Family Registry (CCFR), Genetics and Epidemiology of Colorectal Cancer Consortium (GECCO)

**Affiliations:** 1 Nutrition and Metabolism Branch, International Agency for Research on Cancer, World Health Organization, Lyon, France; 2 Early Cancer Institute, Department of Oncology, School of Clinical Medicine, University of Cambridge, Cambridge, UK; 3 Public Health Sciences Division, Fred Hutchinson Cancer Research Center, Seattle; 4 Division of Epidemiology, Department of Population Health, New York University School of Medicine, New York; 5 Division of Public Health Sciences, Department of Surgery, Washington University School of Medicine, St Louis; 6 Division of Gastroenterology, Department of Medicine, Washington University School of Medicine, St Louis; 7 Alvin J. Siteman Cancer Center, St Louis; 8 Department of Epidemiology and Population Health, Albert Einstein College of Medicine, Bronx, USA; 9 Epigenomics and Mechanisms Branch, International Agency for Research on Cancer, World Health Organization, Lyon, France; 10 Cancer Research UK Cambridge Institute, University of Cambridge, Cambridge, UK; 11 Division of Clinical Epidemiology and Aging Research, German Cancer Research Center (DKFZ), Heidelberg, Germany; 12 Department of Epidemiology, Harvard T.H. Chan School of Public Health, Harvard University, Boston; 13 Department of Medical Oncology, Dana-Farber Cancer Institute, Boston; 14 Program in Molecular Pathological Epidemiology, Department of Pathology, Brigham and Women’s Hospital and Harvard Medical School, Boston; 15 Department of Oncologic Pathology, Dana-Farber Cancer Institute, Boston; 16 Division of Cancer Epidemiology and Genetics, National Cancer Institute, National Institutes of Health, Bethesda, USA; 17 Leeds Institute of Cancer and Pathology, University of Leeds, Leeds, UK; 18 Colorectal Oncogenomics Group, Department of Clinical Pathology, The University of Melbourne, Parkville; 19 University of Melbourne Centre for Cancer Research, Victorian Comprehensive Cancer Centre, Melbourne; 20 Genomic Medicine and Family Cancer Clinic, Royal Melbourne Hospital, Parkville, Australia; 21 Division of Gastroenterology, Massachusetts General Hospital and Harvard Medical School, Boston; 22 Channing Division of Network Medicine, Department of Medicine, Brigham and Women’s Hospital and Harvard Medical School, Boston; 23 Clinical and Translational Epidemiology Unit, Massachusetts General Hospital and Harvard Medical School, Boston, USA; 24 Ontario Health (Cancer Care Ontario), Toronto; 25 Dalla Lana School of Public Health, University of Toronto, Toronto, Canada; 26 Department of Medical Oncology & Therapeutics Research, City of Hope National Medical Center, Duarte, USA; 27 Center for Cancer Research, Medical University of Vienna, Vienna, Austria; 28 Department of Radiation Sciences, Oncology Unit, Umeå University, Umeå; 29 Wallenberg Centre for Molecular Medicine, Umeå University, Umeå, Sweden; 30 Centre for Epidemiology and Biostatistics, Melbourne School of Population and Global Health, The University of Melbourne, Melbourne, Australia; 31 Center for Gastrointestinal Biology and Disease, University of North Carolina, Chapel Hill, USA; 32 Cancer Epidemiology Division, Cancer Council Victoria, Melbourne; 33 Physical Activity Laboratory, Baker Heart and Diabetes Institute, Melbourne, Australia; 34 University of Hawaii Cancer Center, Honolulu, USA; 35 Medical Research Council (MRC) Integrative Epidemiology Unit, Population Health Sciences, Bristol Medical School, University of Bristol, Bristol; 36 Population Health Sciences, Bristol Medical School, University of Bristol, Bristol; 37 National Institute for Health Research (NIHR) Bristol Biomedical Research Centre, University Hospitals Bristol and Weston NHS Foundation Trust and the University of Bristol, Bristol; 38 Department of Colorectal Surgery, North Bristol NHS Trust, Bristol, UK; 39 Cancer Prevention and Control Program, Catalan Institute of Oncology-IDIBELL, L’Hospitalet de Llobregat, Barcelona; 40 CIBER de Epidemiología y Salud Pública (CIBERESP), Madrid; 41 Department of Clinical Sciences, Faculty of Medicine, University of Barcelona, Barcelona, Spain; 42 Division of Human Genetics, Department of Internal Medicine, The Ohio State University Comprehensive Cancer Center, Columbus; 43 Department of Nutrition, Harvard T.H. Chan School of Public Health, Boston, USA; 44 Department of Epidemiology and Biostatistics, School of Public Health, Imperial College London, London, UK; 45 Department of Hygiene and Epidemiology, University of Ioannina School of Medicine, Ioannina, Greece; 46 Department of Molecular Biology of Cancer, Institute of Experimental Medicine of the Czech Academy of Sciences, Prague; 47 Institute of Biology and Medical Genetics, First Faculty of Medicine, Charles University, Prague; 48 Faculty of Medicine and Biomedical Center in Pilsen, Charles University, Pilsen, Czech Republic; 49 Memorial University of Newfoundland, Discipline of Genetics, St. John’s, Canada; 50 Department of Epidemiology, University of Washington, Seattle, USA

**Keywords:** early-onset colorectal cancer, GWAS, genetics, Mendelian randomization, risk factors

## Abstract

**Background:**

The incidence of early-onset colorectal cancer (EOCRC; diagnosed <50 years of age) is rising globally; however, the causes underlying this trend are largely unknown. CRC has strong genetic and environmental determinants, yet common genetic variants and causal modifiable risk factors underlying EOCRC are unknown. We conducted the first EOCRC-specific genome-wide association study (GWAS) and Mendelian randomization (MR) analyses to explore germline genetic and causal modifiable risk factors associated with EOCRC.

**Patients and methods:**

We conducted a GWAS meta-analysis of 6176 EOCRC cases and 65 829 controls from the Genetics and Epidemiology of Colorectal Cancer Consortium (GECCO), the Colorectal Transdisciplinary Study (CORECT), the Colon Cancer Family Registry (CCFR), and the UK Biobank. We then used the EOCRC GWAS to investigate 28 modifiable risk factors using two-sample MR.

**Results:**

We found two novel risk loci for EOCRC at 1p34.1 and 4p15.33, which were not previously associated with CRC risk. We identified a deleterious coding variant (rs36053993, G396D) at polyposis-associated DNA repair gene *MUTYH* (odds ratio 1.80, 95% confidence interval 1.47-2.22) but show that most of the common genetic susceptibility was from noncoding signals enriched in epigenetic markers present in gastrointestinal tract cells. We identified new EOCRC-susceptibility genes, and in addition to pathways such as transforming growth factor (TGF) β, suppressor of Mothers Against Decapentaplegic (SMAD), bone morphogenetic protein (BMP) and phosphatidylinositol kinase (PI3K) signaling, our study highlights a role for insulin signaling and immune/infection-related pathways in EOCRC. In our MR analyses, we found novel evidence of probable causal associations for higher levels of body size and metabolic factors—such as body fat percentage, waist circumference, waist-to-hip ratio, basal metabolic rate, and fasting insulin—higher alcohol drinking, and lower education attainment with increased EOCRC risk.

**Conclusions:**

Our novel findings indicate inherited susceptibility to EOCRC and suggest modifiable lifestyle and metabolic targets that could also be used to risk-stratify individuals for personalized screening strategies or other interventions.

## Highlights

• We conducted GWAS and MR analyses to identify the causes of EOCRC.
• We identified two novel risk loci specific to EOCRC and confirmed the involvement of several known CRC risk variants in EOCRC.
• We also identified new EOCRC risk genes and highlighted the prominent role of pathways such as insulin signaling in EOCRC.
• We identified probable causal associations for body size, fasting insulin, alcohol intake, and educational level with EOCRC.

## Introduction

The incidence rates of colorectal cancer (CRC) in young adults aged <50 years are rising globally, while the incidence rates of CRC in older adults are stable or declining in many of the same countries.^1^ Explanations for the increasing incidence rates of early-onset CRC (EOCRC) are currently lacking.^2^, ^3^, ^4^, ^5^

CRC is a multifactorial disease with high-penetrance genetic syndromes accounting for ∼30% of the EOCRC cases.^6^ Previous genetic studies for EOCRC were limited, focusing on specific germline pathogenic variants.^6^^,^^7^ We previously observed stronger associations between genetic risk scores comprising 95 common CRC single-nucleotide proteins (SNPs) and EOCRC, particularly in the absence of CRC family history.^8^ However, it is currently unknown whether EOCRC has a unique set of genetic susceptibility variants, as a dedicated genome-wide association study (GWAS) for EOCRC with sufficient power to detect genome-wide associations has not been undertaken.

In the United States and several other high-income countries, EOCRC incidence rates have increased in successive birth cohorts since 1950.^9^, ^10^, ^11^ This suggests that higher rates in younger adults may be influenced by changes in lifestyle-related risk factors. However, the role of modifiable risk factors in EOCRC development remains uncertain. Existing evidence is from case–control studies,^12^, ^13^, ^14^ cohort analyses with relatively low case numbers,^15^^,^^16^ or clinical database studies^17^, ^18^, ^19^ that lack high-quality data on many risk factors and covariates. These prior observational studies are also vulnerable to residual confounding and reverse causality, making casual inference challenging. Mendelian randomization (MR), which uses genetic variants as proxies for risk factors to allow causal inference between an exposure and outcome, is largely free from confounding and reverse causality.^20^ To date, MR investigations of associations between modifiable risk factors and EOCRC have not been undertaken.

We carried out a GWAS meta-analysis of EOCRC with 6176 cases and 65 829 controls. Next, using data from this GWAS, we performed two-sample MR analyses to investigate casual associations between 28 potentially modifiable risk factors and EOCRC.

## Patients and methods

### Samples, genotyping, and imputation

**Figure 1:**
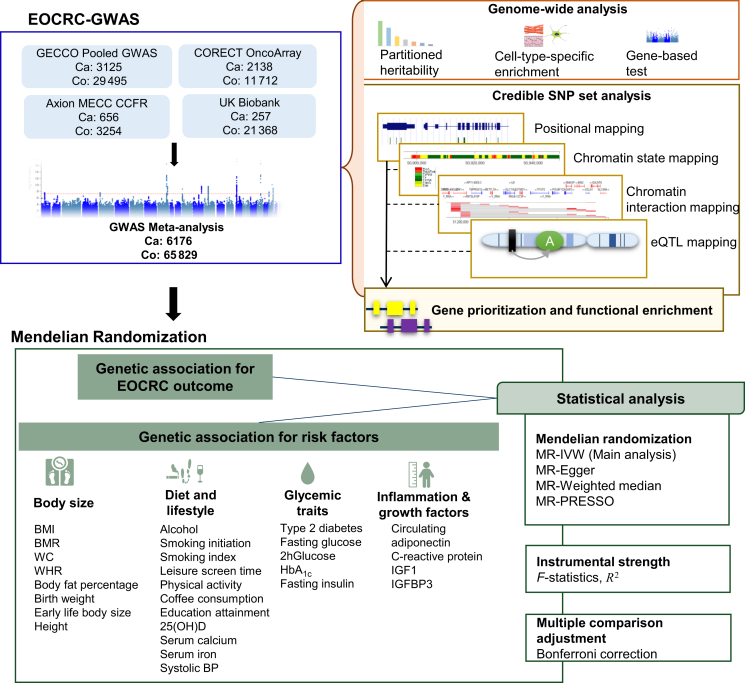
**Study design of the early-onset colorectal cancer (EOCRC) genome-wide association study (GWAS) and Mendelian randomization (MR) analyses.** 25(OH)D, 25-hydroxyvitamin D; 2hGlucose, 2-hour glucose; BMI, body mass index; BMR, basal metabolic rate; eQTL, expression quantitative trait locus; HbA_1c_, glycated hemoglobin; IGF1, insulin-like growth factor 1; IGFBP3, insulin-like growth factor-binding protein 3; IVW, inverse variance-weighted; PRESSO, pleiotropy residual sum and outlier; SNP, single-nucleotide polymorphism; systolic BP, systolic blood pressure; WC, waist circumference; WHR, waist-to-hip ratio.

The overall study design is depicted in Figure 1. The study comprised a meta-analysis of existing genotyped and imputed data for 6176 EOCRC cases (<50 years of age) and 65 829 controls from the Genetics and Epidemiology of Colorectal Cancer Consortium (GECCO), the Colorectal Transdisciplinary Study (CORECT), the Colon Cancer Family Registry (CCFR), and the UK Biobank. The details of the EOCRC cases and controls from each of the studies are presented in Supplementary Materials, available at https://doi.org/10.1016/j.annonc.2024.02.008. Details of genotyping, imputation, and quality control for studies included in the meta-analysis are described previously^21^ and detailed in Supplementary Methods, available at https://doi.org/10.1016/j.annonc.2024.02.008. For the UK Biobank, imputed genotype data were obtained and details of quality control and imputation are described elsewhere.^22^

### Association analysis

The association analysis was performed individually for four datasets: (i) the pooled GECCO dataset including 3135 EOCRC cases and 29 495 controls; (ii) the axiom array dataset with 656 cases and 3254 controls; (iii) the OncoArray dataset with 2138 cases and 11 712 controls; and (iv) the UK Biobank dataset with 257 cases and 21 368 controls. For each of the datasets, imputed dosage with imputation quality *r*^2^ <0.3 and minor allele count (MAC) <50 was used in a logistic regression model adjusted for age, sex, genotyping project, and principal components to adjust for population stratification. Approximate allelic log odds ratio (OR) estimates and standard errors per SNP were calculated, as described previously,^21^ for downstream meta-analysis. An inverse-variance weighted fixed-effects meta-analysis of the aforementioned datasets including 8 910 416 SNPs with minor allele frequency (MAF) >0.5% was implemented in METAL.^23^ The genomic control inflation statics (λ_GC_) was 1.10. To investigate the inflation in genetic signal, we calculated λ_GC_ and linkage disequilibrium score regression (LDSC)^24^ intercept for common variants (MAF ≥1%) overlapping with 1000 Genomes European dataset. The LDSC intercept was 1.05, substantially lower than λ_GC_ of 1.12, indicating that the inflation was mostly due to polygenicity rather than population stratification.

### Genomic risk loci identification, credible SNP set

We used FUMA (version 1.4.1),^25^ a functional mapping and annotation tool, to identify genomic risk loci. FUMA identifies independent variants reaching genome-wide significance (GWAS *P* < 5 × 10^–08^, *r*^2^ = 0.6) and selects lead variants independent from each other at *r*^2^ = 0.1 using 1000 Genomes phase III data for linkage disequilibrium (LD) calculations. By combining LD blocks 500 kb apart, genomic risk loci are defined, often identifying multiple independent significant variants or lead variants at a single genomic risk locus. To identify a credible set of SNPs at each locus, we used the Bayesian false-discovery probability^26^ as described previously^27^ using a prior probability of association of 10^−5^.

### Known risk loci definition

We used the most recent multiethnic CRC GWAS^28^ and searched the NHGRI-EBI Catalog of GWASs until 31 December 2022 to find all CRC associations with a significance level of *P* < 5 × 10^–08^. For multiple studies identifying the same loci, association statistics of the first published GWAS were reported (Supplementary Table S1, available at https://doi.org/10.1016/j.annonc.2024.02.008).

### Sensitivity analysis stratified by high-penetrance gene mutation status

We also conducted a sensitivity analysis on the association of the individual SNPs with EOCRC (individually and through the construction of a genetic risk score) stratified by hereditary syndromes (Lynch) or sporadic case status using two contributing studies [(i) CCFR and (ii) Columbus-area HNPCC Study, OCCPI study, Ohio Colorectal Cancer Prevention Initiative (OSUMC)] which captured this information (more details in the Supplementary Methods, available at https://doi.org/10.1016/j.annonc.2024.02.008).

### Heritability; partitioned and cell-type heritability

We used LDSC to estimate SNP-based heritability (h^2^SNP) and enrichment of functional genomic categories^24^ using precomputed LD scores from 1000 Genomes European data. Also, cell-type group partitioned heritability was estimated using LD scores partitioned across 220 cell-type-specific annotations that were divided into 10 tissue types as described earlier^29^ and detailed in Supplementary Methods, available at https://doi.org/10.1016/j.annonc.2024.02.008.

### Fine mapping and functional genomic annotation of variants

We fine-mapped the credible set of variants at each locus with information on the functional consequences of variants on genes using ANNOVAR^30^; gene body annotations, using GENCODE release 42; Combined Annotation Dependent Depletion (CADD) scores (CADD scores >12.37 suggest a variant is deleterious); Regulome DB scores; 15-core chromatin states representing the accessibility of genomic regions (every 200 bp) from 127 epigenomes in the Roadmap Epigenomics Project^31^; and transcription factor motif binding implemented in HaploReg (version 4.1).^32^ To identify coding variants with predicted functional consequences, we annotated variants with the SIFT^33^ and PolyPhen2^34^ using the SNPnexus version 4^35^ annotation tool.

### Gene-level association and network analyses

We used MAGMA^36^ (implemented in FUMA) for mapping variants to genes. NetworkAnalyst 3.0^37^ was used for protein–protein network analysis using STRING version 10^38^ with a confidence score cut-off of 900 recommended for experimental evidence to support the protein–protein interaction (PPI). Genes with *P* < 0.05 in MAGMA were used as seed genes/proteins. Hub nodes in the interaction map were defined as nodes with degree centrality ≥10. Pathway analysis of the seed proteins identified as hub nodes in the largest subnetwork was conducted using the ‘enrichr’ tool^39^ with the Kyoto Encyclopedia of Genes and Genomes (KEGG) pathway repository.

### Target gene prioritization

We used results of FUMA’s gene prioritization based on (i) positional mapping, which maps SNPs to genes based on physical distance (within a 10-kb window) from known protein-coding genes in the human reference assembly (GRCh37/hg19); (ii) *cis*-quantitative trait loci (eQTL) mapping, which maps SNPs to genes using eQTL data of colorectal datasets from Genotype-Tissue Expression (GTEx)^40^ (sigmoid colon and transverse colon), CEDAR^41^ (rectum and transverse colon), and blood eQTL from BIOS^42^ and eQTLgen^43^ datasets at false discovery rate (FDR) of 0.05; and (iii) chromatin interaction mapping, which maps SNPs to genes using DNA–DNA interaction between the SNP region and a gene region using Hi-C data for the GM12878 lymphoblast cell line. We selected only interaction-mapped genes involving enhancer-promoter regions in colonic and rectal cells from the Roadmap Epigenomics project with an FDR < 1 × 10^–06^ to define significant interactions.^44^ Combining the aforementioned approaches with missense variant annotations from SIFT and Polyphen2 and gene-level results from MAGMA and PPI network hub status, we prioritized putative functional target genes at each genome-wide significant locus.

### Mendelian randomization analyses

We used two-sample MR^45^ to examine associations between 28 potentially modifiable risk factors (all established or suspected risk factors for overall CRC) and EOCRC risk, including eight body size-related traits, 11 diet and lifestyle traits, four inflammatory and growth factors, and five glycemic traits (Figure 1). The largest GWAS or meta-analysis of each risk factor performed until December 2022 was identified. Index SNPs associated with the trait at a *P* value < 5 × 10^–08^ within a 10-Mb window and *r*^2^ < 0.01 were used as instrumental variables (Supplementary Table S2, available at https://doi.org/10.1016/j.annonc.2024.02.008). Exposure genetic instruments were extracted either manually from the respective GWAS or from the Integrative Epidemiology Unit (IEU) OpenGWAS project portal using the TwoSampleMR version 0.5.6 R package (R Foundation, Vienna, Austria).^46^ Effect allele harmonization, MR analyses, and sensitivity analyses were performed using the TwoSampleMR package (version 0.5.6) implemented in R (version 4.2.1). The inverse variance-weighted method was used as the main analytic approach, with MR-Egger,^47^ MR-PRESSO,^48^ and the weighted median method^49^ used as sensitivity analyses to account for pleiotropy. ORs per genetically predicted standard deviation (SD) unit increase were reported for most risk factors to facilitate comparison. A Bonferroni-corrected significance threshold of 0.002 (0.05/28 risk factors) was used to identify associations with strong statistical evidence and *P* values between 0.002 and 0.05 were considered suggestive. Furthermore, we compared overall CRC risk associations for the 28 exposures using summary statistics from the latest CRC GWAS^28^ following similar methods as described earlier.

## Results

### Early-onset colorectal cancer risk loci

**Figure 2:**
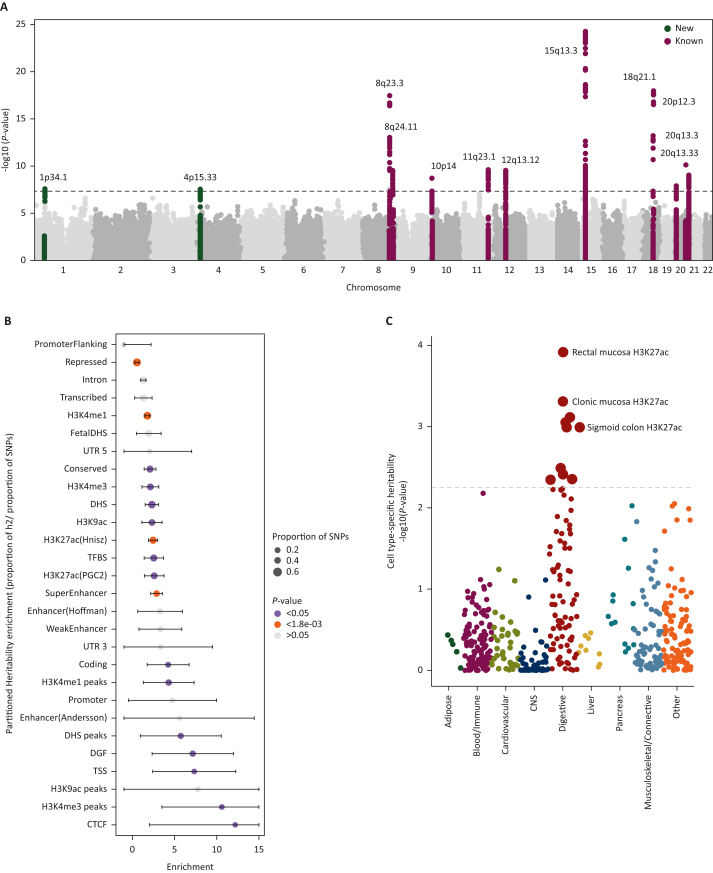
**Early-onset colorectal cancer (EOCRC) genome-wide results**. (A) Manhattan plots displaying the two new and 10 known genome-wide associations between common and rare minor allele frequency > 0.5%, germline genetic variants and EOCRC. The y-axis represents the –log10 values of the meta-analysis *P-*values. (B) Partitioned heritability enrichment estimates for the 28 functional annotations conducted in linkage disequilibrium score regression. (C) Cell-type-specific heritability estimates of EOCRC across different histone marks. CNS, central nervous system; SNP, single-nucleotide polymorphism.

We identified 464 SNPs that attained genome-wide significance (*P* < 5 × 10^–08^) with little evidence of association heterogeneity across the GWAS sets (*P*_het_ > 0.05). LD-based clumping in FUMA mapped these variants to 15 lead SNPs tagging 731 candidate SNPs (LD *r*^2^ > 0.6) within 12 genomic loci >500 kb apart (Figure 2A, Supplementary Table S3, available at https://doi.org/10.1016/j.annonc.2024.02.008). We identified two new loci at 1p34.1 and 4p15.33 that have not been previously associated with CRC, along with 10 previously known risk loci for CRC (Table 1). Three previously identified loci at 11q13.4, 5q22.2, and 15q23 were just below the genome-wide significance (*P* < 4 × 10^–07^) and 106/177 previous risk SNPs were nominally associated with EOCRC risk at *P* < 0.05 (Supplementary Table S1, available at https://doi.org/10.1016/j.annonc.2024.02.008). All 106 SNPs were directionally concordant, and 11 SNPs showed significant heterogeneity (*P* < 0.05) in effect sizes when compared with overall CRC, with generally stronger effect estimates for EOCRC (Supplementary Table S1, available at https://doi.org/10.1016/j.annonc.2024.02.008).

**Table 1:** Summary of the genome-wide significant risk loci for EOCRC represented by the lead SNP in each locus

rsID	Cytoband	Chr	Pos (hg37)	Alt/Risk	RAF	OR (95% CI)	*P*_GWAS_	BFDP	*I*^2^	*P*_het_
**New loci**										
rs186107317	1p34.1	1	46045280	T/A	0.008	1.82 (1.32-1.2.86)	2.35 × 10^–08^	8.16 × 10^–19^	0.0	0.82
rs9991540	4p15.33	4	14881360	G/C	0.09	1.2 (1.14-1.27)	2.28 × 10^–08^	6.66 × 10^–05^	0.0	0.68
**Known loci**										
rs16892766	8q23.3	8	117630683	A/C	0.09	1.33 (1.22-1.45)	3.56 × 10^–18^	1.01 × 10^–24^	0.0	0.68
rs10808556	8q24.21	8	128413147	T/C	0.41	1.14 (1.08-1.19)	3.07 × 10^–10^	7.18 × 10^–06^	39.8	0.17
rs11255835	10p14	10	8732887	C/A	0.45	0.88 (0.84-0.92)	1.82 × 10^–09^	6.42 × 10^–05^	0.0	0.89
rs7944895	11q23.1	11	111167776	G/C	0.30	1.14 (1.1-1.19)	2.60 × 10^–10^	3.06 × 10^–06^	0.0	0.52
rs12427378	12q13.12	12	51074199	T/C	0.34	1.14 (1.09-1.19)	2.76 × 10^–10^	4.31 × 10^–06^	34.0	0.21
rs73376930	15q13.3	15	33012502	A/G	0.21	1.28 (1.20-1.35)	7.05 × 10^–25^	3.39 × 10^–33^	0.0	0.70
rs11874392	18q21.1	18	46453156	T/A	0.45	1.19 (1.15-1.23)	1.27 × 10^–18^	4.66 × 10^–19^	77.7	0.004
rs913245	20p12.3	20	6382301	A/G	0.32	1.12 (1.08-1.18)	1.43 × 10^–08^	0.001	0.0	0.76
rs6066825	20q13.13	20	47340117	A/G	0.38	0.87 (0.84-0.90)	7.13 × 10^–11^	4.64 × 10^–07^	0.0	0.40
rs2427291	20q13.33	20	60921324	G/A	0.20	0.85 (0.8-0.9)	9.69 × 10^–10^	2.81 × 10^–06^	0.0	0.78

Alt, alternative/other allele; BFDP, Bayesian false-discovery probability; Chr, chromosome; CI, confidence interval; EOCRC, early-onset colorectal cancer; GWAS, genome-wide association study; *I*^2^, proportion of the total variation due to heterogeneity; OR, odds ratio calculated for risk allele; *P*_GWAS_, *P-*value from GWAS meta-analysis; *P*_het_, *P-*value for heterogeneity across studies; Pos, base position; RAF, risk allele frequency; Risk, risk allele; SNP, single-nucleotide polymorphism.

As hereditary cases with high-penetrance genetic mutations could not be systematically removed, we conducted a sensitivity analysis on a smaller subset of cases from the CCFR and OSUMC studies that have data on Lynch and other high-penetrance rarer genetic CRC syndromes (*N* = 202). Overall, a similar pattern of GWAS effect estimates was found according to Lynch syndrome status for most SNPs (all Phets > 0.05), albeit with wider confidence intervals (CIs) because of limited power due to the smaller sample size. For three SNPs (rs11255835, rs12427378, and rs2427291), however, the estimates were attenuated toward the null (Supplementary Figure S1, available at https://doi.org/10.1016/j.annonc.2024.02.008). Similar estimates were also obtained when combined into a genetic risk score (Lynch cases per unit increase, OR 1.59, 95% CI 1.05-2.42; *P* = 0.03) and non-Lynch cases per unit increase (OR 2.99, 95% CI 2.73-3.27; *P* = 2.12 × 10^–121^; Phet = 0.55; Supplementary Table S4, available at https://doi.org/10.1016/j.annonc.2024.02.008).

### Heritability of EOCRC and cell-type-specific enrichment

The narrow sense heritability of EOCRC was estimated to be 6.2% (standard error 0.009). Heritability enrichment of genome functional categories found enrichment in regions with high levels of active transcription, such as H3K27ac regions/peaks (enrichment = 2.45, *P* = 9.5 × 10^–08^), H3K9ac regions (enrichment = 1.77, *P* = 1.5 × 10^–05^), and in super-enhancers (enrichment = 2.87, *P* = 3.03 × 10^–07^; Figure 2B, Supplementary Table S5, available at https://doi.org/10.1016/j.annonc.2024.02.008). Partitioned heritability across cell-type-specific epigenetic marks identified strong enrichment in histone marks in gastrointestinal epithelial cells (Figure 2C, Supplementary Table S6, available at https://doi.org/10.1016/j.annonc.2024.02.008. These results are consistent with previous GWAS of other traits where SNP trait heritability was shown to be enriched in transcriptionally active open chromatin regions in trait-relevant cell types.^50^^,^^51^

### Functional enrichment of EOCRC-risk SNPs

To further fine map variants, we identified 570 credible sets of SNPs across the 12 loci using a Bayesian false-discovery probability cut-off of <0.1 (Supplementary Table S7A, available at https://doi.org/10.1016/j.annonc.2024.02.008). Four loci had exonic variants (Supplementary Table S7B, available at https://doi.org/10.1016/j.annonc.2024.02.008); however, the credible SNPs were mostly intronic and intergenic and overlapped with regulatory regions, particularly active transcription sites and enhancers (Supplementary Figures S2A, B, and S3A–J, available at https://doi.org/10.1016/j.annonc.2024.02.008) that are enriched in gastrointestinal tract epithelial cells (Supplementary Figure S4, available at https://doi.org/10.1016/j.annonc.2024.02.008).

**Figure 3:**
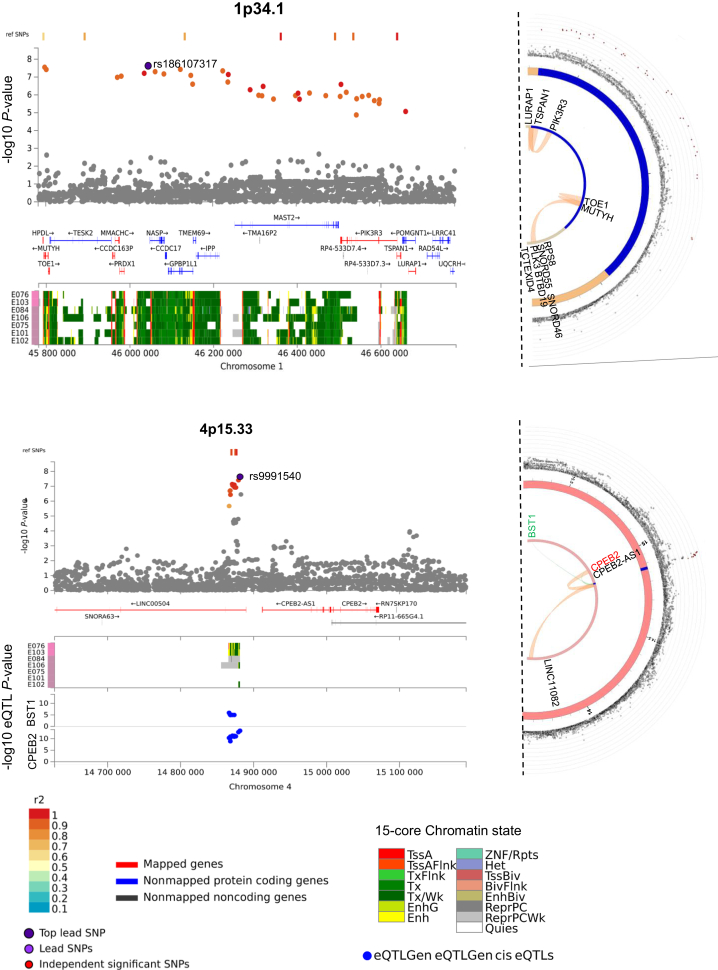
**Regional plots of the two new early-onset colorectal cancer risk loci.** The genome-wide association study meta-analysis −log10 *P-*values (y-axis) of the single-nucleotide polymorphisms (SNPs) are shown according to their chromosomal positions (x-axis) based on GRCh37 in the main panel. The extent of linkage disequilibrium with the top SNP is denoted by the color scheme from gray (*r*^2^ < 0.1) to dark red (*r*^2^ = 1.0), with *r*^2^ estimated from EUR 1000 Genomes data. The lower panel shows the 15-core chromatin states from the Roadmap Epigenomics project (E075, colonic mucosa; E076, colon smooth muscle; E106, sigmoid colon; E101, rectal mucosa 1; E102, rectal mucosa 2; E103, rectal smooth muscle; E084, fetal large intestine). The lowermost panel shows the –log10 *P-*values from the expression quantitative trait locus (eQTL) analysis where available. The semicircular plot on the right shows Hi-C chromatin interaction involving the credible SNP set in the loci from the GM12878 cell line. The genes in green represent eQTL-mapped genes, black represents chromatin interaction-mapped, and red are both eQTL and chromatin interaction-mapped genes at each locus.

*Cis*-regulatory transcriptional networks of the credible SNPs identified 428 *cis*-eQTLs at FDR <0.05 in multiple datasets. We found eQTLs at 8 of 10 previously known CRC risk loci, as well as at the 4p15.33 locus for the *BST1* and *CPEB2* genes (Supplementary Table S8, available at https://doi.org/10.1016/j.annonc.2024.02.008). Around 13.7% of the credible set of SNPs mapped to regions with significant (FDR < 1 × 10^–06^) chromatin interactions. In gastrointestinal epithelial cells, we identified three significant chromatin interactions at 1p34.1, between enhancer containing rs36053993 and promoter regions of multiple genes at two loci, and between rs145667118 and rs41309177 overlapping enhancers and promoter regions of the *PIK3R3*, *TSPAN1,* and *LUPAP1* genes. At 4p15.33, we observed significant interactions between eight enhancer-overlapping SNPs and *CPEB2*, *CPEB2-AS1,* and long intergenic noncoding RNA *LINC01182* (Supplementary Table S9, available at https://doi.org/10.1016/j.annonc.2024.02.008, Figure 3). We further confirmed 22 other significant interactions at eight previously known CRC risk loci (Supplementary Table S9, available at https://doi.org/10.1016/j.annonc.2024.02.008, Supplementary Figure S5A–F, available at https://doi.org/10.1016/j.annonc.2024.02.008).

### Gene-level association and protein–protein interaction networks

Using MAGMA-based gene-level association tests, we identified 16 genes at genome-wide significance level (*P* < 2.6 × 10^–06^) involved in transforming growth factor (TGF) β signaling, mothers against decapentaplegic (SMAD) binding, BMP, and mismatch repair pathways (Supplementary Figure S6A, Supplementary Tables S10 and S11, available at https://doi.org/10.1016/j.annonc.2024.02.008). To obtain a more inclusive functional overview, we performed a PPI network analysis using genes with MAGMA *P* < 0.05 as ‘seeds’ and obtained a large subnetwork with 165 seed proteins as major hub nodes (Supplementary Figure S6B, available at https://doi.org/10.1016/j.annonc.2024.02.008). These included known CRC-associated genes such as *MYC*, *TCF7L2*, *SMAD3*, *EIF3H,* and *PIK3R3* at the newly identified locus 1p34.1. *CPEB2* and *MUTYH* at the new loci were also part of the subnetwork (Supplementary Table S12, available at https://doi.org/10.1016/j.annonc.2024.02.008). This is in line with the observation that trait-associated genes are often part of larger biological networks.^51^^,^^52^ The seed hub proteins were enriched for cancer and immune-related pathways, cellular processes—such as cell cycle, apoptosis, and DNA repair—and CRC risk factors such as insulin resistance and type 2 diabetes (Supplementary Table S13, available at https://doi.org/10.1016/j.annonc.2024.02.008). The enrichment of several pathways involved in infection might reflect shared cellular signaling between cancer and infection, particularly related to inflammation and immune response.^53^

### Functional gene prioritization of EOCRC

**Figure 4:**
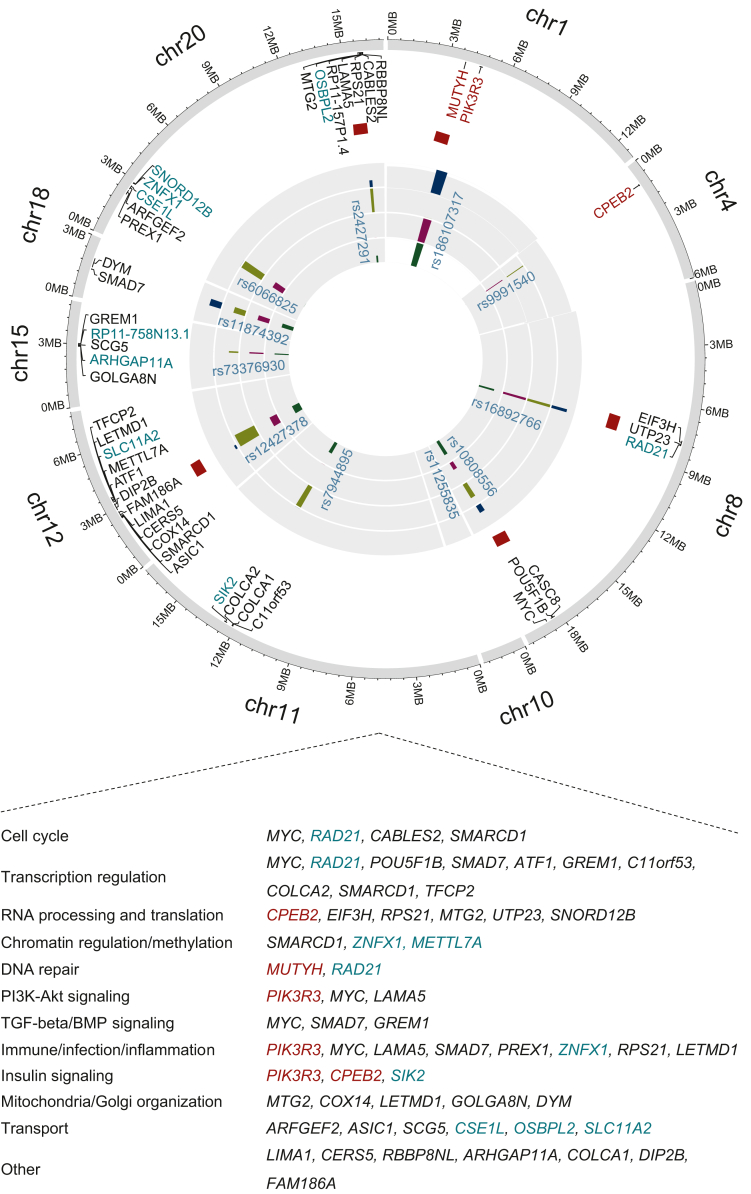
**Summary of prioritized candidate genes associated with early-onset colorectal cancer risk.** Shown are the 44 candidate genes identified in this study and gene-level functional annotation from the Gene Ontology database^79^ and literature search. Genes in burgundy are the genes identified in the two newly identified loci; gene names in black are previously prioritized genes in known loci, and green-cyan genes are the newly identified genes in known risk loci. Red blocks in front of gene names represent genes with nonsynonymous coding variants, dark green bars represents positional mapping; dark magenta bars represent chromatin-interaction; dark blue bars represents protein-protein interaction hubs, and light green bars represents eQTL mapping. Akt, protein kinase B; BMP, bone morphogenetic protein; eQTL, expression quantitative trait loci; PI3K, phosphatidylinositol kinase; PPI, protein–protein interaction; TGF, transforming growth factor.

We identified potential genes based on functional fine-mapping including deleterious nonsynonymous classification, eQTL and chromatin interaction data, gene-based tests, and hub status in PPI networks (Supplementary Table S14, available at https://doi.org/10.1016/j.annonc.2024.02.008). At each locus, genes nominated by the maximum of these approaches were selected with additional weightage to deleterious coding, eQTL genes, and genes previously identified in CRC-GWAS.^21^^,^^27^^,^^28^^,^^54^ Notably, some genes at the known risk loci were not previously associated with CRC risk, including the DNA repair gene *RAD21* involved in loss of heterozygosity and Wnt signaling in CRC^55^; and genes such as *SIK2*, *TFCP2*, *ARHGAP11A*, *ZNFX1*, *SNORD12B*, *CSE1L,* and *OSBPL2* (Figure 4), all with reported oncogenic roles in several gastrointestinal malignancies.^56^, ^57^, ^58^, ^59^, ^60^, ^61^, ^62^

Among the new loci, at 1p34.1 we identified the DNA repair gene *MUTYH* with a rare (MAF 0.8%) nonsynonymous variant (rs36053993, G396D) associated with an increased risk of EOCRC (OR 1.80, 95% CI 1.47-2.22; *P* = 2.84 × 10^–08^). With 6176 cases, we had ∼70% power to detect the association in a one-stage study (Supplementary Figure S8, available at https://doi.org/10.1016/j.annonc.2024.02.008). This biallelic *MUTYH* variant is associated with adenomatous polyposis of the colon^63^ and an increased risk of CRC at younger ages.^64^ Fine mapping of the locus also identified *PIK3R3* encoding the regulatory subunit p55 of phosphatidylinositol kinase (PI3K) that is known to promote cell proliferation in CRC by inducing the epithelial-to-mesenchymal transition^65^ and the p53/CDKN1A (p21) signaling pathway.^66^ At 4p15.33, we prioritized the translational regulatory factor *CPEB2*,^67^ which is known to promote senescence and suppress epithelial-to-mesenchymal transition by regulating p53, HIF1α, and Twist1 translation.^68^^,^^69^ Knockdown of *CPEB in vitro* caused p53 protein levels to decrease by 50%.^70^ The risk variants at this locus were *cis*-eQTLs, downregulating CPEB2 expression (Supplementary Table S8, available at https://doi.org/10.1016/j.annonc.2024.02.008). In The Cancer Genome Atlas (TCGA) and GTEx datasets, the expression of *CPEB2* was lower in CRC cells compared with normal cells (Supplementary Figure S8, available at https://doi.org/10.1016/j.annonc.2024.02.008), suggesting that these SNPs might increase the EOCRC risk by lowering *CPEB2* expression and affecting p53 translation and cellular senescence.

The prioritized genes annotated to several common biological processes/pathways based on gene-level functional annotation from the Gene Ontology (GO) database^71^ and literature search. This includes common cellular processes, such as cell cycle, DNA repair, transcription, translation, and chromatin regulation; CRC signaling pathways such as PI3K/protein kinase B (AKT), BMP, TGFβ; and immune- and inflammation-related pathways. Three of the newly identified target genes, *CPEB2*, *PIK3R3*, and *SIK2*, had roles in insulin signaling and several others were involved in organelle membrane or intracellular transport (Figure 4).

### Mendelian randomization

**Figure 5:**
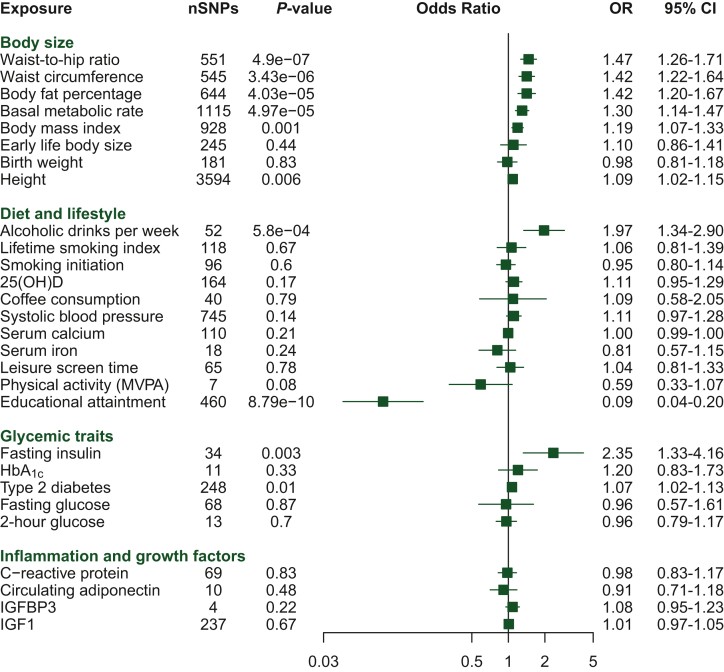
**Mendelian randomization (MR) analyses.** Odds ratios (ORs) from inverse variance-weighted MR analysis for the association between putative risk factors and early-onset colorectal cancer (EOCRC). All associations are expressed as OR per standard deviation (SD) increase in the risk factor except for alcoholic drinks per week and fasting insulin, which were expressed as OR per unit increase in the natural logarithm of the exposures. For categorical risk factors such as smoking initiation (ever versus never), type 2 diabetes (yes versus no), and physical activity (inactive versus active), the ORs were expressed as unit change in the exposure, compared with the reference group. 25(OH)D, 25-hydroxyvitamin D; HbA_1c_, glycated hemoglobin; IGF, insulin growth factor; MVPA, moderate-to-vigorous physical activity; SNP, single-nucleotide polymorphism.

Genetically predicted body size measures were positively associated with EOCRC risk, with the highest OR estimates observed for central adiposity measurements such as waist-to-hip ratio (OR per 0.1 increase (1–SD) 1.47, 95% CI 1.26-1.71) and waist circumference [OR per 13.4 cm increase (1–SD) 1.42, 95% CI 1.22-1.64). BMI, body fat percentage, and basal metabolic rate were also positively associated with EOCRC risk. No significant association was found between genetically predicted birth weight and early-life body size with EOCRC risk. We observed a suggestive positive association between genetically predicted adult height and EOCRC risk [OR per 9.2 cm increase (1–SD) 1.09, 95% CI 1.03-1.16; Figure 5).

Among diet and lifestyle factors, genetically predicted per unit increase in log-transformed alcoholic drinks per week was strongly associated with EOCRC risk (OR per unit increase 1.97, 95% CI 1.34-2.90). Smoking; coffee consumption; leisure screen time; and blood concentrations of vitamin D, calcium, and iron were not associated with EOCRC risk. Genetically predicted higher years of schooling were strongly associated with lower EOCRC risk (Figure 5).

For glycemic traits, we observed a positive association per unit increase in log(pmol/l) for fasting insulin levels (OR 2.35, 95% CI 1.33-4.16). A suggestive positive association was observed with type 2 diabetes, but the MR-Egger sensitivity analysis effect estimate was unsupportive of a causal effect (Figure 5).

Genetic instruments for these potentially modifiable risk factors were 4 to 3594 SNPs. *F*-statistics were high (>10), indicating strong instruments, for all considered traits (Supplementary Tables S2 and S15, available at https://doi.org/10.1016/j.annonc.2024.02.008). Similar patterns of effect estimates were observed for EOCRC and overall CRC (Supplementary Figure S9, available at https://doi.org/10.1016/j.annonc.2024.02.008). While alcohol consumption was more strongly associated with EOCRC, lifetime smoking index and physical activity were more clearly associated with overall CRC. Overall, weighted median and MR-Egger sensitivity analyses showed similar magnitude and effect direction in causal estimates for body size parameters, alcohol consumption, and fasting insulin measures (Supplementary Table S16 & Supplementary Figure S10, available at https://doi.org/10.1016/j.annonc.2024.02.008). Leave-one-out analyses for inverse variance-weighted tests did not identify any bias from single-sensitive SNPs for any of the significant associations (Supplementary Table S17, available at https://doi.org/10.1016/j.annonc.2024.02.008). MR-Egger regression did not identify any evidence of horizontal pleiotropy for most exposures, and similar estimates were found when the few outliers detected by MR-PRESSO were excluded from analyses (Supplementary Tables S18 and S19, available at https://doi.org/10.1016/j.annonc.2024.02.008).

## Discussion

We report the first comprehensive GWAS for EOCRC. We identified new EOCRC risk loci, confirmed the involvement of previously identified CRC risk loci, and report new EOCRC-susceptibility genes and pathways through functional annotation. We identified a high penetrance deleterious coding variant and showed that most of the EOCRC genetic susceptibility comes from the noncoding signals that are enriched in epigenetic markers present in the epithelial cells of the gastrointestinal tract. Our findings show that common germline variants alone are unlikely to explain a substantial heritability or account for the increase in EOCRC incidence.

Our study provides novel insights into possible biological mechanisms underlying EOCRC. Alongside known CRC susceptibility pathways such as TGFβ, Wnt, SMAD, BMP, and PI3K signaling, which are crucial for maintaining normal intestinal homeostasis,^21^^,^^27^^,^^28^ we highlight the role of insulin signaling, immune, and infection-related pathways in EOCRC. Intestinal insulin signaling is critical for maintaining normal epithelial integrity, and damage to the intestinal barrier causes gut dysbiosis, leading to inflammation and an increased risk of developing colon cancer.^72^^,^^73^ Target genes with immune function, by contrast, might act through various mechanisms that affect immune surveillance, chronic inflammation, host–pathogen interactions, and the tumor microenvironment.^53^ However, given the relatively smaller size of the current GWAS compared with the overall CRC study,^28^ several important genes and pathways likely remain unidentified. We could only explain 6.2% of the SNP-based heritability of EOCRC, highlighting the need for larger GWASs and whole genome sequencing studies to identify the missing heritability and provide further biological insights into EOCRC susceptibility.

The current GWAS enabled us to explore potential causal relationships between EOCRC and modifiable risk factors in comprehensive MR analyses. Temporal increases in exposures such as obesity, unhealthy diets, and other unfavorable lifestyle behaviors in young adults over the past few decades have been linked to the increase in the incidence of early-onset cancers.^74^^,^^75^ However, disentangling the causal relevance of each individual exposure in traditional observational studies is challenging because of confounding and potential bias from reverse causality. In our MR analyses, we found novel evidence of potential causal associations for higher levels of body size and metabolic factors—such as body fat percentage, waist circumference, waist-to-hip ratio, basal metabolic rate, and fasting insulin—higher alcohol drinking, and lower education attainment with increased EOCRC risk.

The positive effect estimates we observed for adiposity are consistent with results from some observational studies^15^^,^^76^ and an increasing obesity trend in young adults.^77^ Hyperinsulinemia and insulin resistance are frequently present in individuals who are obese. The positive effect estimate we observed for fasting insulin and EOCRC is consistent with recent evidence supporting a role for metabolic dysregulation in EOCRC development.^17^

Given that per capita alcohol consumption increased between 1960 and 2010 in many countries^78^ and the candidate risk factor status of alcohol for EOCRC in observational studies,^14^^,^^75^ our findings suggest a probable causal association between alcohol drinking and EOCRC risk. This is in contrast to a weaker and statistically nonsignificant association we and others have found for overall CRC.^79^ Interestingly, alcohol intake has been associated with CRC tumors exhibiting LINE-1 hypomethylation,^80^ a key feature of EOCRC tumors.^81^^,^^82^ Additional studies investigating the effects of different patterns of early-life alcohol consumption (e.g. moderate and binge drinking) are needed to further probe the alcohol–EOCRC relationship. Overall, our MR results suggest that public health policies to reduce obesity and alcohol consumption might have a positive impact on EOCRC prevention. Further, pharmacological or lifestyle interventions that lower circulating insulin levels may be beneficial in preventing EOCRC.

We also observed a strong inverse effect estimate for genetically predicted higher years of schooling with EOCRC risk, a result directionally consistent with what we and others found for overall CRC, and possibly a consequence of socioeconomic status and related behavioral risk factors.^83^

Our study has several notable strengths. In addition to being the first dedicated GWAS of EOCRC conducted with substantial power and detailed functional analyses of the identified genetic associations, this was the first comprehensive MR analysis to understand potentially modifiable risk factors of EOCRC. We conducted multiple sensitivity analyses to account for potential biases due to pleiotropy, and our results remained generally robust across these analyses. However, some MR analyses may have been limited in statistical power, and the size of the EOCRC GWAS limited our ability to carry out analysis stratified by sex and tumor location.^84^ Because of the lack of data on high-penetrance gene mutations in several contributing studies, we were unable to systematically account for genetic mutations related to Lynch and other rarer hereditary cancer syndromes in our GWAS analysis. However, sensitivity analysis on a subset of cases with Lynch data showed a similar pattern of effect estimates, suggesting that our EOCRC GWAS meta-analysis and MR analyses are largely representative of sporadic disease which is driving the alarming rising incidence rates in young adults globally.^2^^,^^4^^,^^5^ Certain risk factors such as alcohol, education attainment, and fasting insulin showed relatively large effect sizes, which might be indicative of either stronger associations with EOCRC compared with overall CRC or some inflation due to a smaller sample size. Overall, for our MR analyses, the genetic instruments used were obtained from a single timepoint which means that for time-varying exposures, temporal effects could not be inferred.^85^ Furthermore, the exposure and outcome GWASs were conducted mostly on individuals of European descent, which restricted the testing of applicability to other at-risk populations. Nonetheless, this provides further support for the prioritization of future large-scale multiethnic studies.

In conclusion, our findings provide novel insights into the inherited susceptibility to EOCRC including target genes and functional pathways that provide insights into the biological basis of EOCRC. It also reveals key modifiable targets for primary prevention, such as excess adiposity, hyperinsulinemia, and alcohol drinking. Our findings may help prioritize individuals for personalized screening regimens or other intervention strategies.
