# Supplementary material for: Genome-wide association studies and Mendelian randomization analyses provide insights into the causes of early-onset colorectal cancer
Source: Ann Oncol. 2024 Jun;35(6):523–36. doi: 10.1016/j.annonc.2024.02.008 (PMC11213623; doi:10.1016/j.annonc.2024.02.008)
Supplement: Supplementary Figures [file mmc1.pdf]

SUPPLEMENTARY NOTE

Contents:

Supplementary methods
Details of the EOCRC cases and controls
Supplementary Figures
~~SUF 1~~: Association of lead SNPs at 12 significant genomic loci stratified by Lynch status in CCFR and OSMUC
~~SUF 2A~~: Enrichment of the credible SNP set for genomic annotations
~~SUF 2B:~~ Overlap of the credible SNP set for different functional chromatin states.
~~SUF 3A-J~~: Regional plots showing the top and individual lead SNPs and the LD structure of the known GWAS significant loci.
~~SUF 4~~: Enrichment of the credible SNP set for histone marks in different cell-types
~~SUF 5A-F~~: Plots for hi-C chromatin interaction involving the credible SNP set in each loci from the GM12878 cell line.
~~SUF6A~~: Manhattan plot for gene-level test in MAGMA
~~SUF6B~~: Protein-protein interaction map of genes with P<0.05 in MAGMA
~~SUF 7~~: Statistical power calculation to detect OR of 1.8 with 0.8% MAF in a one-stage study
~~SUF 8:~~ Comparison of the expression of the prioritized genes in colorectal cancer tissues and normal colonic cells from TCGA and GTEx datasets.
~~SUF 9~~: Scatter plot of the beta estimates from exposure and outcome GWAS for the tested risk factors
~~SUF10~~: Odds ratios from inverse variance weighted MR-analysis for association between putative risk factors and overall CRC
Funding
Acknowledgement

Supplementary Methods:

Genotyping and imputation:
We performed sample and variant level QC on the genotyped data as follows: samples with missing call rate ≥3%, samples with discrepancies between reported and genotypic sex, close second-degree relatives, and individuals of non-European ancestry were removed. Variants with missing call rate >2% or Hardy-Weinberg equilibrium (HWE) P<1e-04 were also removed. To identify population outliers, we performed principal component analysis (PCA). This was followed by phasing and imputation using minimac3 and/or the Haplotype Reference Consortium (HRC) panel (using the University of Michigan Imputation Server) after pooling studies that used very similar genotyping platforms.

Replication in cases with Lynch syndrome:
Screening of CRC cases for the presence of Lynch syndrome was systematically carried out for two studies, namely, Colon Cancer Family Registry (CCFR) and Columbus-area HNPCC Study, OCCPI study, Ohio Colorectal Cancer Prevention Initiative (OSUMC) as described previously (Archambault et al, 2020). For OSUMC, all cases were tested for mismatch repair deficiency using immunohistochemistry. Cases with Lynch syndrome-like characteristics were subjected to additional genetic testing in order to definitively diagnose Lynch syndrome based on the presence of one or more germline high-penetrance mutations in DNA mismatch repair genes (MLH1, MSH2, MSH6, PMS2) or the EPCAM gene. In these studies, we used logistic regression to evaluate the association between individual lead SNPs, genetic risk scores and EOCRC risk.

Genetic Risk Score (GRS) Computation:
GRS was calculated for 12 SNPs, one from each of the two newly identified loci and the ten previously identified EOCRC risk loci (rs186107317, rs9991540, rs16892766, rs10808556, rs11255835, rs7944895, rs12427378, rs73376930, rs11874392, rs913245, rs6066825, rs2427291), as follows:

$$\mathbf{GRS_j = \sum_{k=1}^{12} w_k x_{jk}} \,,$$

where, GRS_j is the risk score for individual j, x_{jk} is the number of risk alleles for the k-th variant and w_k is the weight [ln(OR)] of the k-th variant. We computed logistic regression models using GRS as continuous variable and as tertiles, comparing the highest with the lowest tertile. All models were adjusted for sex, age and 20 principal components.

Partitioned and cell type heritability
We used stratified LDSC to determine whether any of the 28 functional genomic categories were enriched for contribution to the h2SNP relative to the proportion of variants annotated to that category. We estimated cell-type group partitioned heritability using LD scores partitioned across 220 cell-type-specific annotations that were divided into 10 tissue types (central nervous system, cardiovascular, kidney, adrenal/pancreas, gastrointestinal, connective/bone, immune/hematopoietic, skeletal muscle, liver, and other) implemented in LDSC. Cell-type-specific annotations included H3K9ac, H3K27ac, H3K4me1, and H3K4me3 enhancer and promoter specific histone marks in each tissue types.

Mendelian randomization analysis:
We used Inverse variance-weighted Mendelian randomization (IVW-MR) as the main analysis. Under the assumption of all valid instrumental variables (IVs), IVW-MR estimates the effect of the risk factor on the disease outcome using a weighted average of the effect estimates from each genetic variant, where the weights are based on the inverse of the variance of the effect estimates. Further to detect and correct for biases arising from horizontal pleiotropy, we used an MR-Egger regression method that uses a linear regression model to estimate the causal effect of the exposure on the outcome, while also testing for the presence of horizontal pleiotropy by assessing the deviation of the slope of the regression line. We also computed OR estimates using

the complementary weighted-median method that can give valid MR estimates under the presence of horizontal pleiotropy when up to 50% of the included instruments are invalid. Also, the MR-PRESSO (Mendelian Randomization Pleiotropy RESidual Sum and Outlier) distortion test was used to estimate if horizontal pleiotropy caused by any identified outlier SNPs biased the effect estimates ($P < .05$).

Mendelian randomization analysis: Exposure phenotype definition

Birth Weight: Weight at birth in grams, measured at birth or self-reported at baseline/follow-up (Warrington et al. (2019), PMID: 31043758).
Body Mass Index: Calculated at baseline from measured height and weight. (kg/m2) (Yengo et al. (2018), PMID: 30124842).
Basal Metabolic Rate: Calculated at baseline using a body composition analyzer based on Dual-energy X-ray absorptiometry using bioelectrical impedance analysis (in Kilojoule) (https://gwas.mrcieu.ac.uk, GWAS -ID: ukb-b-16446).
Waist to Hip Ratio: Calculated by dividing waist circumference by hip circumference measured at baseline (Pulit et al. (2019), PMID: 30239722).
Waist Circumference: Measured in cm at baseline (in cm) (https://gwas.mrcieu.ac.uk/, GWAS-ID: ukb-b-9405).
Body Fat Percentage: Estimated at baseline using whole-body bio-impedance measures using the body composition analyzer (in %) (https://gwas.mrcieu.ac.uk, GWAS-ID: ukb-b-8909).
Early-life Body Size: Estimated at baseline from categorical self-reported perceived body size at age 10, using the questionnaire as follows: "When you were 10 years old, compared to average would you describe yourself as thinner, plumper, or about average?" (Richardson et al. (2020), PMID: 32376654).
Height: Height in cm either measured, self reported or from electronic health records (Yengo et al. (2022), PMID: 36224396).
Type 2 Diabetes: Study specific T2D definitions summerised as: diagnostic fasting glucose, 2-hour plasma glucose or HbA1c levels, hospital discharge diagnosis, use of oral diabetes medication, electronic health records or self report (Mahajan et al. (2018), PMID: 30297969).
Fasting Glucose (FG), 2-Hour Glucose (2hGlu), Fasting Insulin (FI), glycated hemoglobin (HbA1c): Data for FG and 2hGlu were measured in mmol/l, FI measured in pmol/l, and HbA1c in % [where possible, studies reported HbA1c as a National Glycohemoglobin Standardization Program (NGSP) percent]. Similar to previous MAGIC efforts, individuals were excluded if they had type 1 or type 2 diabetes (defined by physician diagnosis); reported use of diabetes-relevant medication(s); or had a FG ≥7 mmol/L, 2hGlu ≥11.1mmol/L, or HbA1c ≥ 6.5%. 2hGlu measures were obtained 120 minutes after a glucose challenge in an oral glucose tolerance test (OGTT). Measures for FG and FI taken from whole blood were corrected to plasma level using the correction factor 1.1380 (Chen et al. (2021), PMID: 34059833).
Smoking Initiation: In GSCAN , calculated as a binary phenotype (Liu M et al. (2019), PMID: 30643251).
1. Any participant reporting ever being a regular smoker in their life (current or former) were coded "2", while any participant who reported never being a regular smoker in their life were coded "1".
2. Does not include information about pipes/cigar/chew, or other non-cigarette forms of tobacco use.
3. This phenotype was measured in a variety of ways.
a. Have you smoked over 100 cigarettes over the course of your life?
b. Have you ever smoked every day for at least a month?
c. Have you ever smoked regularly?
Lifetime Smoking Index (CSI): Estimated following the method outlined by Leffondré, Abrahamowicz, Xiao, and Siemiatycki (2006) by combining the smoking measures into a lifetime smoking index along with a simulated half-life (τ) constant. Half-life captures the exponentially decreasing effect of smoking at a given time on health outcomes. The value of half-life was determined by simulating the effects of lifetime smoking on lung cancer and overall mortality in the UK Biobank. Both suggested the best fitting value as 18.

These values were used to fit the final model which is:tsc* = max(tsc - δ, 0)
dur* = max(dur + tsc - δ, 0) – tsc*
lifetime smoking = (1 – $0.5^{dur*/\tau}$) ($0.5^{tsc*/\tau}$) ln(int+1)
…where τ = half-life, δ = lag time, int = cigarettes per day, tss = time started smoking, tsc = time since cessation, dur = duration of smoking (either age-tss for current smokers or [age-tsc]-tss for former smokers). So, in our case, where δ = 0, tsc* = tsc and consequently. Values of lifetime smoking were treated as continuous in subsequent analysis (Wootton et al. (2019), PMID: 31689377).

Alcoholic drinks per week: 1. In GSCAN, defined as the average number of drinks a participant reported drinking each week, aggregated across all types of alcohol. If a study recorded binned response ranges (e.g., 1-4 drinks per week, 5-10 drinks per week) it used the midpoint of the range. For example, if an individual reported 1-5 drinks per week, it assumed they drank 2.5 drinks per week on average.
2. This was measured in a variety of ways.
a. In the past week, how many alcoholic beverages did you have?
b. Thinking about the past year, on the average how many drinks did you have each week?
3. This phenotype was left-anchored at 1 and log-transformed prior to analysis, in order to prevent outliers from having undue leverage on analyses.
(Liu M. (2019), PMID: 30643251).

Coffee consumption: Estimated as cups/day in UK Biobank derived from "How many cups of coffee do you drink each DAY? (Include decaffeinated coffee) (https://gwas.mrcieu.ac.uk/, GWAS ID: ukb-b-5237).

Serum Calcium: Total calcium concentrations in serum were determined in UKB using a colorimetric method on the Beckman Coulter AU5800 during Initial assessment visit (2006-2010) at which participants were recruited and consent given (Sinnott-Armstrong N, et al. PMID: 33462484).

25(OH)D: In UK Biobank, vitamin D 25OHD levels were measured in blood samples collected at two instances: the initial assessment visit, conducted between 2006 and 2010, and a repeat assessment visit, conducted between 2012 and 2013 using the Diasorin Liason, a chemiluminescent immunoassay (CLIA) was used for the quantitative determination of 25OHD. The GWAS was conducted on UKB participants mostly from the initial assessment visit (99.6%) (Revez et al. (2020), PMID: 32242144).

Serum iron: Non-fasting or fasting serum iron was measured using FerroZine calorimetric method. For individuals with multiple measurements, the initial measurement was used in the analyses (Moksnes et al. (2022), PMID: 35710628).

Physical activity (MVPA), Leisure screen time (LST): Self-reported moderate-to-vigorous intensity physical activity during leisure time (MVPA) and leisure screen time (LST) were determined using study/cohort specific questionnaires as detailed by Wang et al. (2022), (PMID: 36071172).

Education attainment: Education attainment was measured as number of years of schooling completed (*EduYears*). EduYears was measured at an age of at least 30 and the phenotype was constructed by mapping each major educational qualification that can be identified from the cohort’s survey measure to an International Standard Classification of Education (ISCED) category and imputing a years-of-education equivalent for each ISCED category (Lee et al. (2018), PMID:30038396).

Systolic blood pressure (SBP): The mean SBP values at baseline were calculated from two sets of automated or manual BP measurements. The average of the manual and automated BP measurements were considered for individuals who had both types of measurements. Single value was used for individuals who had only one available BP measurement (Evangelou et al, (2018), PMID: 30224653).

Circulating Adiponectin: Adiponectin levels were measured using ELISA or RIA methods at baseline for either population-based, family-based, or case-control studies (Dastani et al. (2012), PMID: 22479202).

C-reactive Protein (CRP): Baseline serum CRP was measured in mg/L by using standard laboratory techniques and transformed the values by natural log. Individuals with auto-immune diseases, individuals taking immune-modulating agents (if this information was available), and individuals with CRP amounts 4 SD or more away from the mean were excluded from all GWAS analyses (Lighart et al, (2018), PMID: 30388399).

IGF1: IGF1 in serum were determined in UKB using CLIA analysis on a DiaSorin Ltd. LIASON XL during Initial assessment visit (2006-2010) at which participants were recruited and consent given (Sinnott-Armstrong N et al. (2021), PMID: 33462484).

IGFBP3: IBFBP3 levels were estimated by various cohort-specific methods as detailed by Teumer A et al. (2016). Levels were estimated on either fasting/non-fasting plasma or serum using either or a combination of chemiluminescence assay, ELISA, RIA or immunoradiometric methods (Teumer A et al. (2016),PMID: 27329260).

Details of the EOCRC cases included in the study

Study	Country/Continent	Cases			
		Total	Female	Male	Age Mean (SD)
ASTERISK	France	33	12 (36.4)	21	F: 45.2 (2.5) M: 45.6 (2.4)
ATBC	Finland	0			F: NA (NA) M: NA (NA)
CCFR	USA, Canada, Australia	1965	1042 (53)	923	F: 42.1 (5.7) M: 42.7 (5.8)
CGN	USA	94	44 (46.8)	50	F: 38.7 (9.1) M: 43.2 (4.4)
CLUEII	USA	41	21 (51.2)	20	F: 43 (5.4) M: 43.1 (4.3)
Colo23	USA	10	4 (40)	6	F: 45.5 (1.9) M: 44.2 (4.4)
ColoCare	USA, Germany	72	24 (33.3)	48	F: 41.4 (6.2) M: 42.3 (5.9)
COLON	Netherlands	34	18 (52.9)	16	F: 44.3 (5) M: 42.4 (5.2)
CORSA	Austria	255	85 (33.3)	170	F: 43 (5.4) M: 44.4 (4.5)
CPSII	USA	1	1 (100)	0	F: 49 (NA) M: NA (NA)
CRCGEN	Spain	54	23 (42.6)	31	F: 42.7 (7.5) M: 44.9 (3.9)
CzechCCS	Czech Republic	163	71 (43.6)	92	F: 41.2 (7) M: 41.5 (6.4)
DACHS	Germany	166	72 (43.4)	94	F: 44.2 (4.4) M: 44.5 (4)
DALS	USA	104	49 (47.1)	55	F: 42.9 (5.2) M: 43.3 (5.6)
EDRN	USA	43	19 (44.2)	24	F: 42.1 (5.9) M: 43.8 (5.1)
EPIC	Europe	369	203 (55)	166	F: 44.6 (4.2) M: 44.6 (4.2)
EPICOLON	Spain	62	33 (53.2)	29	F: 41.7 (6.6) M: 43.6 (5.5)
ESTHER_VERDI	Germany	8	4 (50)	4	F: 42.2 (6.6) M: 47 (2.3)
FIRE3	Germany, Austria	23	8 (34.8)	15	F: 45.8 (4.1) M: 44.9 (4.8)
GALEON	Spain	2	2 (100)	0	F: 48 (1.4) M: NA (NA)
HawaiiCCS	USA	7	3 (42.9)	4	F: 46 (1) M: 44 (2.6)
HispanicCCS	USA	1	1 (100)	0	F: 31 (NA) M: NA (NA)
HPFS	USA	48	0 (0)	48	F: NA (NA) M: 48.5 (0.7)
Kentucky	USA	120	62 (51.7)	58	F: 43.3 (6.3) M: 44.3 (5.4)
LCCS	UK	66	31 (47)	35	F: 43.6 (5.2) M: 44.7 (5.2)
MAVERICC	USA	33	20 (60.6)	13	F: 43.8 (5.9) M: 41.8 (4.5)
MCCS	Australia	32	17 (53.1)	15	F: 44.1 (2.9) M: 45.1 (3)
MEC	USA	33	13 (39.4)	20	F: 46.9 (1.6) M: 46.9 (1.4)
MECC	Isreal	329	190 (57.8)	139	F: 42.7 (6.3) M: 42.1 (6.5)
MOFFITT	USA	26	13 (50)	13	F: 44.9 (5.9) M: 43.4 (4.9)
MSKCC	USA	20	15 (75)	5	F: 42.1 (7.1) M: 41.6 (7.2)
NCCCSI	USA	19	7 (36.8)	12	F: 45.6 (3.2) M: 45.8 (2.5)
NCCCSII	USA	86	35 (40.7)	51	F: 45.7 (2.9) M: 46.1 (2.8)
NGCCS	Germany	135	78 (57.8)	57	F: 42.9 (4.8) M: 43.3 (6)
NHS	USA	157	157 (100)	0	F: 47 (1.9) M: NA (NA)
NHSII	USA	109	109 (100)	0	F: 36.5 (4.5) M: NA (NA)
NSHDS	Sweden	69	37 (53.6)	32	F: 44.5 (5.7) M: 43.1 (6.3)
OSUMC	USA	574	259 (45.1)	315	F: 42.9 (5.8) M: 42.4 (6.1)
PHS	USA	64	0 (0)	64	F: NA (NA) M: 45.6 (2.8)
PLCO	USA	0			F: NA (NA) M: NA (NA)
PMH-CCFR	USA	0			F: NA (NA) M: NA (NA)
PPS3	USA	8	2 (25)	6	F: 44.5 (2.1) M: 46.7 (1.8)
PPS4	USA	8	2 (25)	6	F: 47.5 (2.1) M: 47.8 (1.3)
PURIFICAR	Puerto Rico, USA	15	9 (60)	6	F: 43.4 (7.2) M: 46.5 (1.2)
SEARCH	UK	256	133 (52)	123	F: 43.3 (5.8) M: 43.5 (5.2)
SELECT	USA	0			F: NA (NA) M: NA (NA)
SLRCCS	Sweden	131	63 (48.1)	68	F: 43.7 (5.3) M: 43.4 (5)
SMC_COSM	Sweden	18	5 (27.8)	13	F: 49 (0) M: 47.2 (1)
SMS	USA	3	1 (33.3)	2	F: 38 (NA) M: 47 (1.4)
TRIBE	Italy	53	25 (47.2)	28	F: 43.7 (4.4) M: 43.8 (5.1)
UKB	UK	257	139 (54.1)	118	F: 45.8 (2.7) M: 46 (2.6)
USC_HRT_CRC	USA	0			F: NA (NA) M: NA (NA)
VITAL	USA	0			F: NA (NA) M: NA (NA)
WHI	USA	0			F: NA (NA) M: NA (NA)

Details of the tumor anatomical subsite of EOCRC cases included in the study

Study	Proximal	Distal	Rectal	Unknown
ASTERISK	8	14	10	1
ATBC	0	0	0	0
CCFR	540	532	756	137
CGN	0	0	0	94
CLUEII	15	13	12	1
Colo23	3	2	4	1
ColoCare	0	0	36	36
COLON	5	10	11	8
CORSA	44	78	51	82
CPSII	0	0	1	0
CRCGEN	14	16	20	4
CzechCCS	3	12	27	121
DACHS	28	52	86	0
DALS	51	47	0	6
EDRN	13	11	19	0
EPIC	92	111	137	29
EPICOLON	6	28	21	7
ESTHER_VERDI	1	3	3	1
FIRE3	0	0	0	23
GALEON	1	0	1	0
HawaiiCCS	2	4	1	0
HispanicCCS	0	0	0	1
HPFS	7	6	5	30
Kentucky	45	58	0	17
LCCS	8	20	34	4
MAVERICC	0	0	0	33
MCCS	7	11	14	0
MEC	12	7	10	4
MECC	57	106	132	34
MOFFITT	6	4	16	0
MSKCC	0	0	0	20
NCCCSI	7	8	2	2
NCCCSII	0	33	24	29
NGCCS	3	5	8	119
NHS	19	24	15	99
NHSII	0	0	0	109
NSHDS	20	22	22	5
OSUMC	142	159	234	39
PHS	17	15	25	7
PLCO	0	0	0	0
PMH-CCFR	0	0	0	0
PPS3	0	0	0	8
PPS4	0	0	0	8
PURIFICAR	0	0	0	15
SEARCH	65	74	112	5
SELECT	0	0	0	0
SLRCCS	30	37	54	10
SMC_COSM	5	7	6	0
SMS	0	0	0	3
TRIBE	0	0	0	53
UKB	58	75	102	22
USC_HRT_CRC	0	0	0	0
VITAL	0	0	0	0
WHI	0	0	0	0
Total	1334	1604	2011	1227

Details of the EOCRC controls included in the study

Study	Country/Continent	Controls			
		Total	Female	Male	Age Mean (SD)
ASTERISK	France	946	423 (44.7)	523	F: 62.7 (10.5) M: 61.3 (9.8)
ATBC	Finland	32	0 (0)	32	F: NA (NA) M: 57.1 (4.3)
CCFR	USA, Canada, Australia	3001	1511 (50.3)	1490	F: 54.6 (12.2) M: 56 (11.7)
CGN	USA	0			F: NA (NA) M: NA (NA)
CLUEII	USA	252	135 (53.6)	117	F: 61.7 (12.1) M: 60.3 (10.7)
Colo23	USA	124	54 (43.5)	70	F: 65.6 (10.5) M: 64.5 (11.4)
ColoCare	USA, Germany	38	21 (55.3)	17	F: 52.1 (11.4) M: 59.4 (14.5)
COLON	Netherlands	697	259 (37.2)	438	F: 60.8 (6.9) M: 62.1 (6.2)
CORSA	Austria	1949	753 (38.6)	1196	F: 61.7 (11.9) M: 58.4 (12.5)
CPSII	USA	347	182 (52.4)	165	F: 68 (6.1) M: 69.2 (5.2)
CRCGEN	Spain	1022	495 (48.4)	527	F: 62.6 (11.8) M: 66.1 (10.3)
CzechCCS	Czech Republic	1616	734 (45.4)	882	F: 50.2 (14.7) M: 50.9 (12.5)
DACHS	Germany	2788	1094 (39.2)	1694	F: 68.6 (10.8) M: 68.4 (10.2)
DALS	USA	1163	526 (45.2)	637	F: 64.3 (9.8) M: 63.6 (10)
EDRN	USA	312	160 (51.3)	152	F: 59 (10.4) M: 60 (10.7)
EPIC	Europe	2317	1239 (53.5)	1078	F: 56.6 (8) M: 56.8 (8.1)
EPICOLON	Spain	341	142 (41.6)	199	F: 58.7 (6.6) M: 59.8 (6.4)
ESTHER_VERDI	Germany	436	153 (35.1)	283	F: 65.1 (7.2) M: 64.9 (5.9)
FIRE3	Germany, Austria	0			F: NA (NA) M: NA (NA)
GALEON	Spain	0			F: NA (NA) M: NA (NA)
HawaiiCCS	USA	530	191 (36)	339	F: 61.9 (8.8) M: 60.5 (7.7)
HispanicCCS	USA	0			F: NA (NA) M: NA (NA)
HPFS	USA	989	0 (0)	989	F: NA (NA) M: 65.1 (9.4)
Kentucky	USA	1132	572 (50.5)	560	F: 66.7 (6.6) M: 60.4 (9.2)
LCCS	UK	683	362 (53)	321	F: 67.7 (9) M: 68.5 (7.8)
MAVERICC	USA	0			F: NA (NA) M: NA (NA)
MCCS	Australia	212	99 (46.7)	113	F: 60.1 (7.5) M: 60.7 (7.3)
MEC	USA	429	202 (47.1)	227	F: 63.5 (7.8) M: 62.7 (8.4)
MECC	Isreal	3851	1850 (48)	2001	F: 70 (12.5) M: 71.2 (11.6)
MOFFITT	USA	0			F: NA (NA) M: NA (NA)
MSKCC	USA	0			F: NA (NA) M: NA (NA)
NCCCSI	USA	464	204 (44)	260	F: 66.1 (9.5) M: 66.1 (9.6)
NCCCSII	USA	686	258 (37.6)	428	F: 63.4 (10.7) M: 64.6 (9.1)
NGCCS	Germany	0			F: NA (NA) M: NA (NA)
NHS	USA	1890	1890 (100)	0	F: 61.4 (8.4) M: NA (NA)
NHSII	USA	101	101 (100)	0	F: 36.3 (4.2) M: NA (NA)
NSHDS	Sweden	414	261 (63)	153	F: 57.7 (7.7) M: 53.4 (7.3)
OSUMC	USA	2436	1476 (60.6)	960	F: 51.4 (15) M: 53.4 (14.4)
PHS	USA	387	0 (0)	387	F: NA (NA) M: 58.6 (8.9)
PLCO	USA	2687	1077 (40.1)	1610	F: 62.6 (5.3) M: 63.6 (5.3)
PMH-CCFR	USA	118	118 (100)	0	F: 61.8 (7.4) M: NA (NA)
PPS3	USA	452	186 (41.2)	266	F: 55.6 (9.9) M: 56.7 (9)
PPS4	USA	910	377 (41.4)	533	F: 56.3 (6.1) M: 58.2 (6.6)
PURIFICAR	Puerto Rico, USA	74	48 (64.9)	26	F: 53.5 (10.9) M: 58.5 (12.5)
SEARCH	UK	1149	1016 (88.4)	133	F: 36.5 (15.7) M: 54.8 (7.6)
SELECT	USA	268	0 (0)	268	F: NA (NA) M: 65.8 (6.6)
SLRCCS	Sweden	2493	1177 (47.2)	1316	F: 58.3 (15.7) M: 59.6 (15.3)
SMC_COSM	Sweden	856	366 (42.8)	490	F: 63.7 (8.2) M: 63.7 (8.1)
SMS	USA	127	71 (55.9)	56	F: 58.7 (11.2) M: 63.2 (11)
TRIBE	Italy	0			F: NA (NA) M: NA (NA)
UKB	UK	21368	9064 (42.4)	12304	F: 59.3 (7.2) M: 60.4 (6.7)
USC_HRT_CRC	USA	400	400 (100)	0	F: 63.9 (6.8) M: NA (NA)
VITAL	USA	282	136 (48.2)	146	F: 67.3 (6.1) M: 66 (6.4)
WHI	USA	3060	3060 (100)	0	F: 67.3 (6.7) M: NA (NA)

~~SUF 1~~: Association of lead SNPs at 12 significant genomic loci stratified by Lynch status in CCFR and OSMUC

SNPs	Cytoband	Chr	Pos	Alt/Risk	Cases	P	OR	95%-CI
**rs186107317**								
Lynch syndrome	1p34.1	1	46045280	T/A	202	0.27	1.80	[0.63; 5.11]
Non-Lynch syndrome					2337	7.32e-10	1.92	[1.56; 2.37]
**rs9991540**								
Lynch syndrome	4p15.33	4	14881360	G/C	202	0.45	1.14	[0.81; 1.58]
Non-Lynch syndrome					2337	5.78e-07	1.18	[1.11; 1.26]
**rs16892766**								
Lynch syndrome	8q23.3	8	117630683	A/C	202	0.35	1.17	[0.84; 1.62]
Non-Lynch syndrome					2337	1.31e-15	1.30	[1.22; 1.38]
**rs10808556**								
Lynch syndrome	8q24.21	8	128413147	T/C	202	0.04	1.24	[1.01; 1.52]
Non-Lynch syndrome					2337	6.95e-11	0.88	[0.84; 0.91]
**rs11255835**								
Lynch syndrome	10p14	10	8732887	C/A	202	0.64	1.05	[0.85; 1.29]
Non-Lynch syndrome					2337	5e-09	0.88	[0.85; 0.92]
**rs7944895**								
Lynch syndrome	11q23.1	11	111167776	G/C	202	0.21	0.87	[0.71; 1.08]
Non-Lynch syndrome					2337	4.3e-11	0.87	[0.83; 0.91]
**rs12427378**								
Lynch syndrome	12q13.12	12	51074199	T/C	202	0.95	0.99	[0.81; 1.22]
Non-Lynch syndrome					2337	1.39e-13	1.17	[1.12; 1.21]
**rs73376930**								
Lynch syndrome	15q13.3	15	33012502	A/G	202	0.05	1.27	[1.00; 1.61]
Non-Lynch syndrome					2337	9.28e-26	1.28	[1.23; 1.34]
**rs11874392**								
Lynch syndrome	18q21.1	18	46453156	T/A	202	0.44	0.92	[0.76; 1.13]
Non-Lynch syndrome					2337	3.15e-19	0.83	[0.80; 0.87]
**rs913245**								
Lynch syndrome	20p12.3	20	6382301	A/G	202	0.31	0.90	[0.73; 1.10]
Non-Lynch syndrome					2337	3.28e-08	0.89	[0.85; 0.93]
**rs6066825**								
Lynch syndrome	20q13.13	20	47340117	A/G	202	0.16	0.86	[0.69; 1.06]
Non-Lynch syndrome					2337	7.42e-09	0.88	[0.85; 0.92]
**rs2427291**								
Lynch syndrome	20q13.33	20	60921324	G/A	202	0.61	1.07	[0.83; 1.37]
Non-Lynch syndrome					2337	1.38e-10	0.84	[0.80; 0.89]

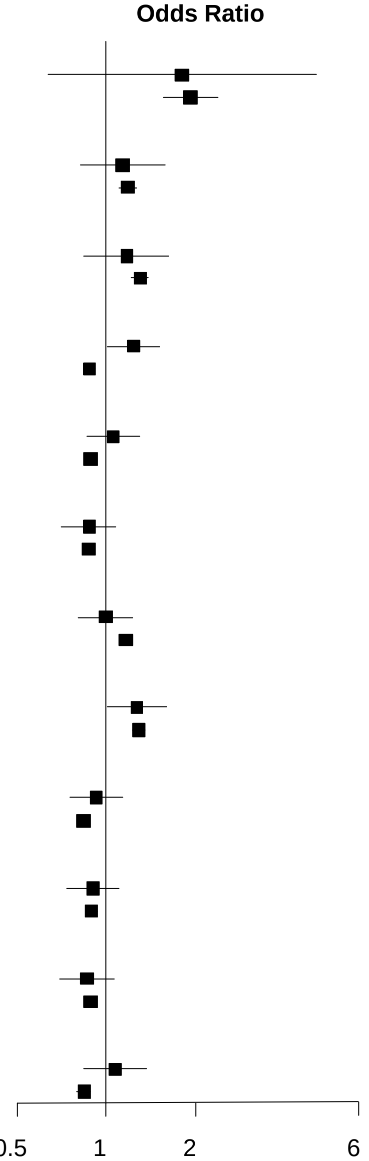

All P-heterogeneity (Lynch vs. Non-Lynch) > 0.05

SUF 2A:Enrichment of the credible SNP set for genomic annotations

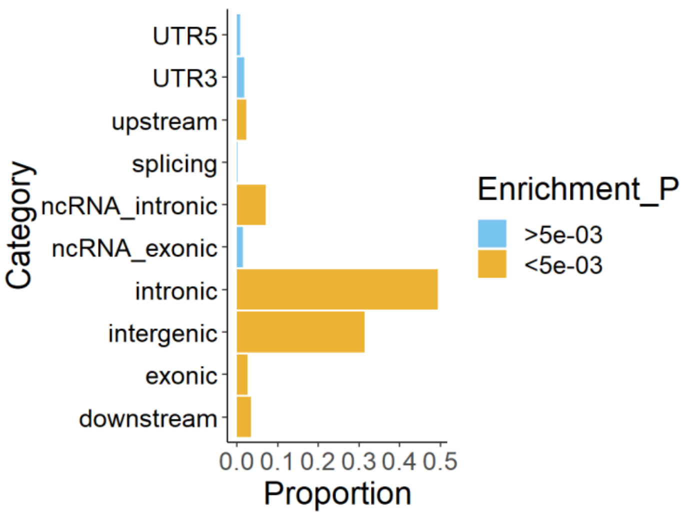

SUF 2B: Overlap of the credible SNP set for different functional chromatin states.

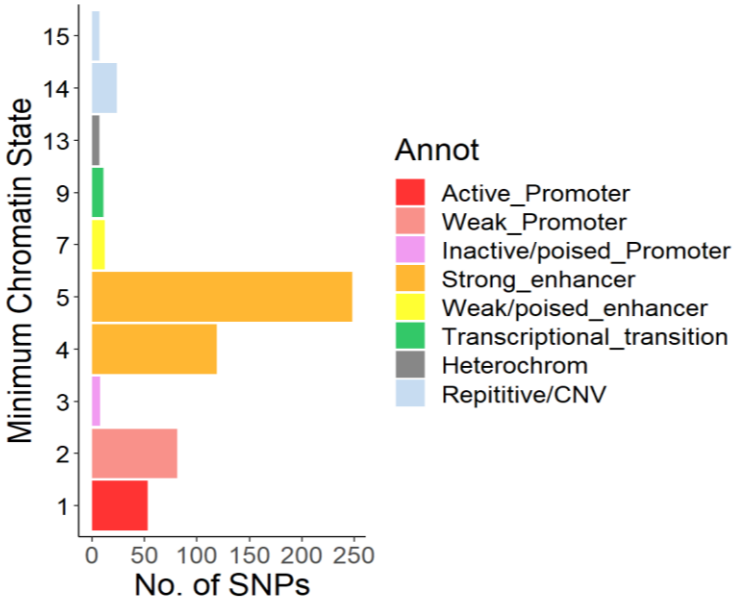

Supplementary figure ~~SUF 3A-J~~: Regional plots showing the top and individual lead SNPs and the LD structure of the known GWAS significant loci.

(A)

8q23.3

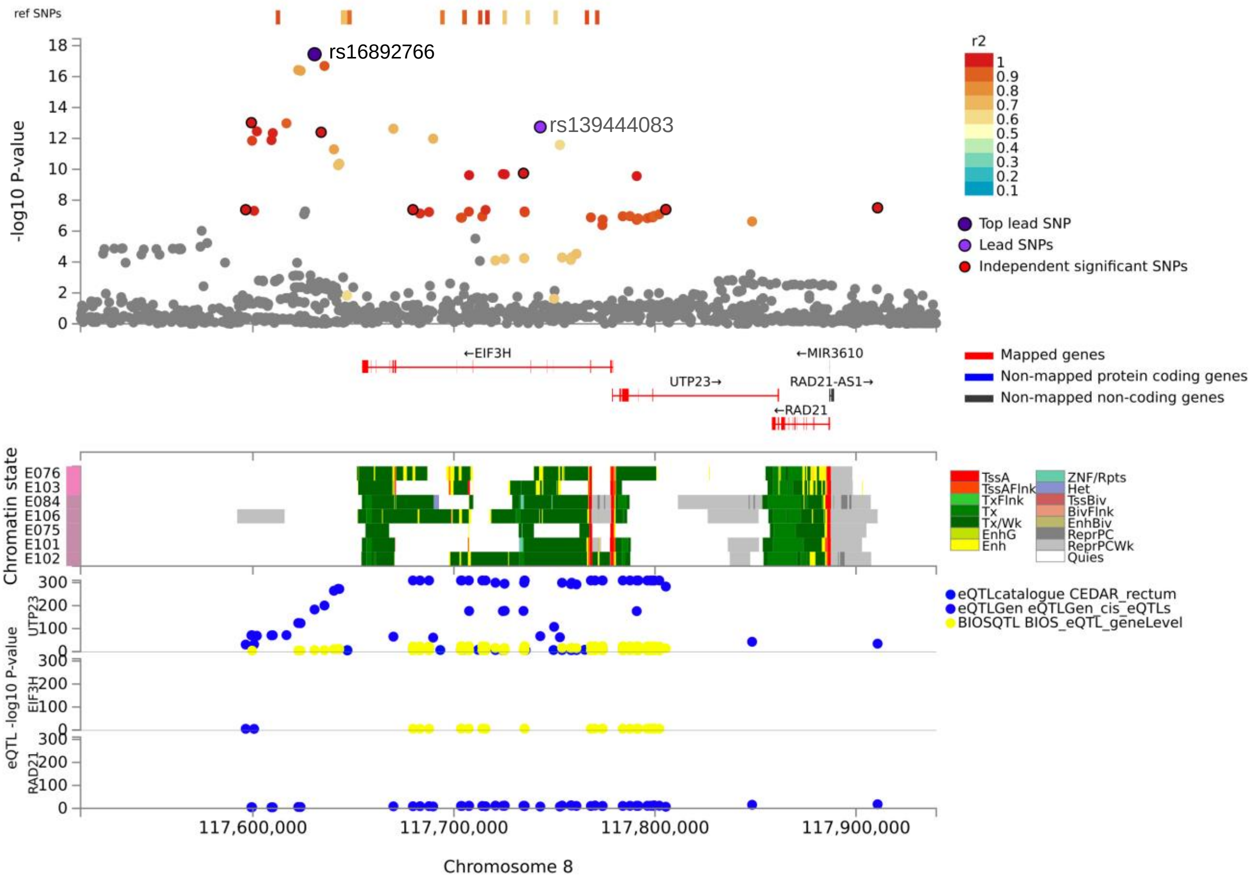

(B)

8q24.21

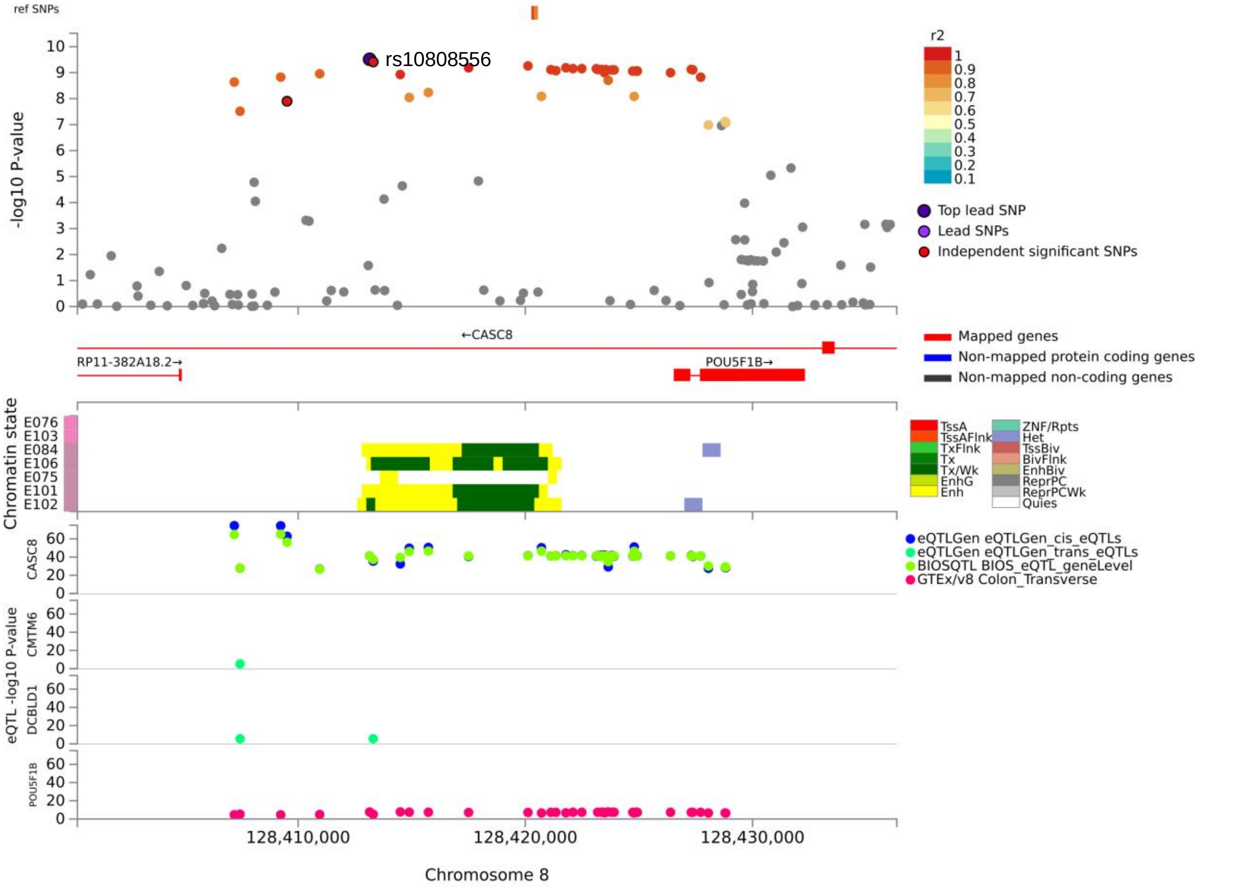

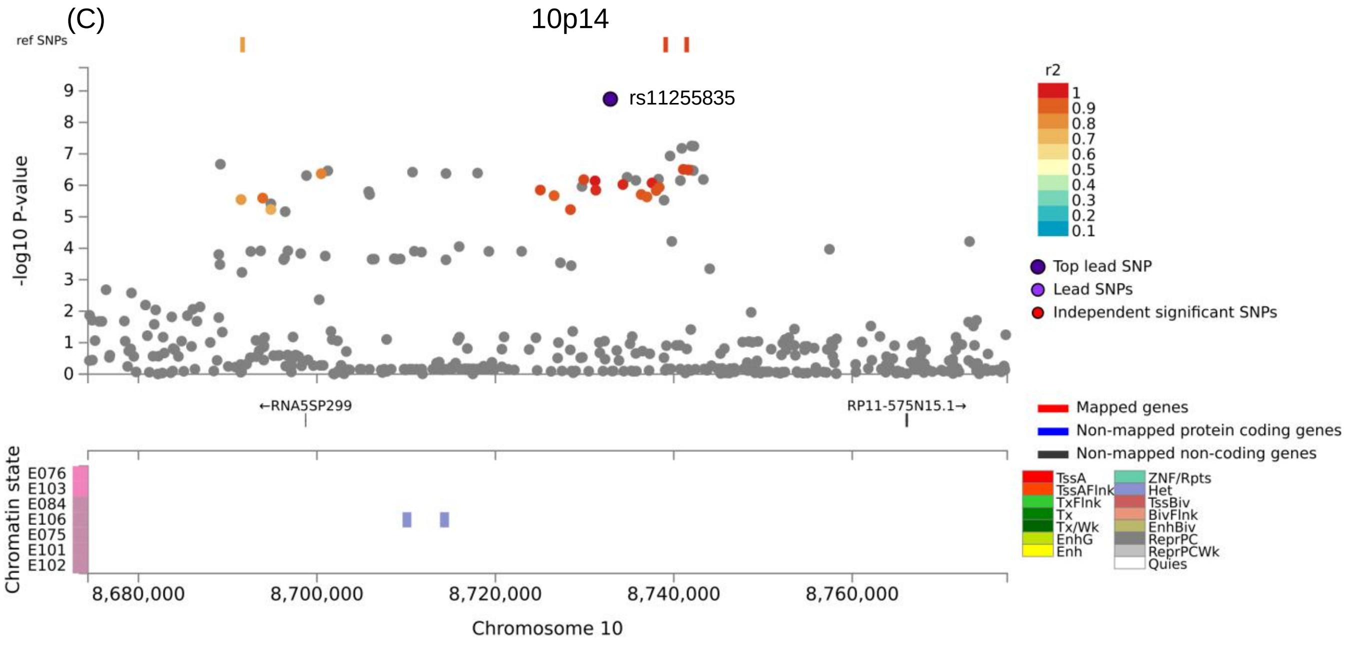
(C)
10p14
ref SNPs
rs11255835
-log10 P-value
r2
Top lead SNP
Lead SNPs
Independent significant SNPs
←RNA5SP299
RP11-575N15.1→
Mapped genes
Non-mapped protein coding genes
Non-mapped non-coding genes
Chromatin state
E076
E103
E084
E106
E075
E101
E102
TssA
TssAFlnk
TxFlnk
Tx
Tx/Wk
EnhG
Enh
ZNF/Rpts
Het
TssBiv
BivFlnk
EnhBiv
ReprPC
ReprPCWk
Quies
8,680,000
8,700,000
8,720,000
8,740,000
8,760,000
Chromosome 10

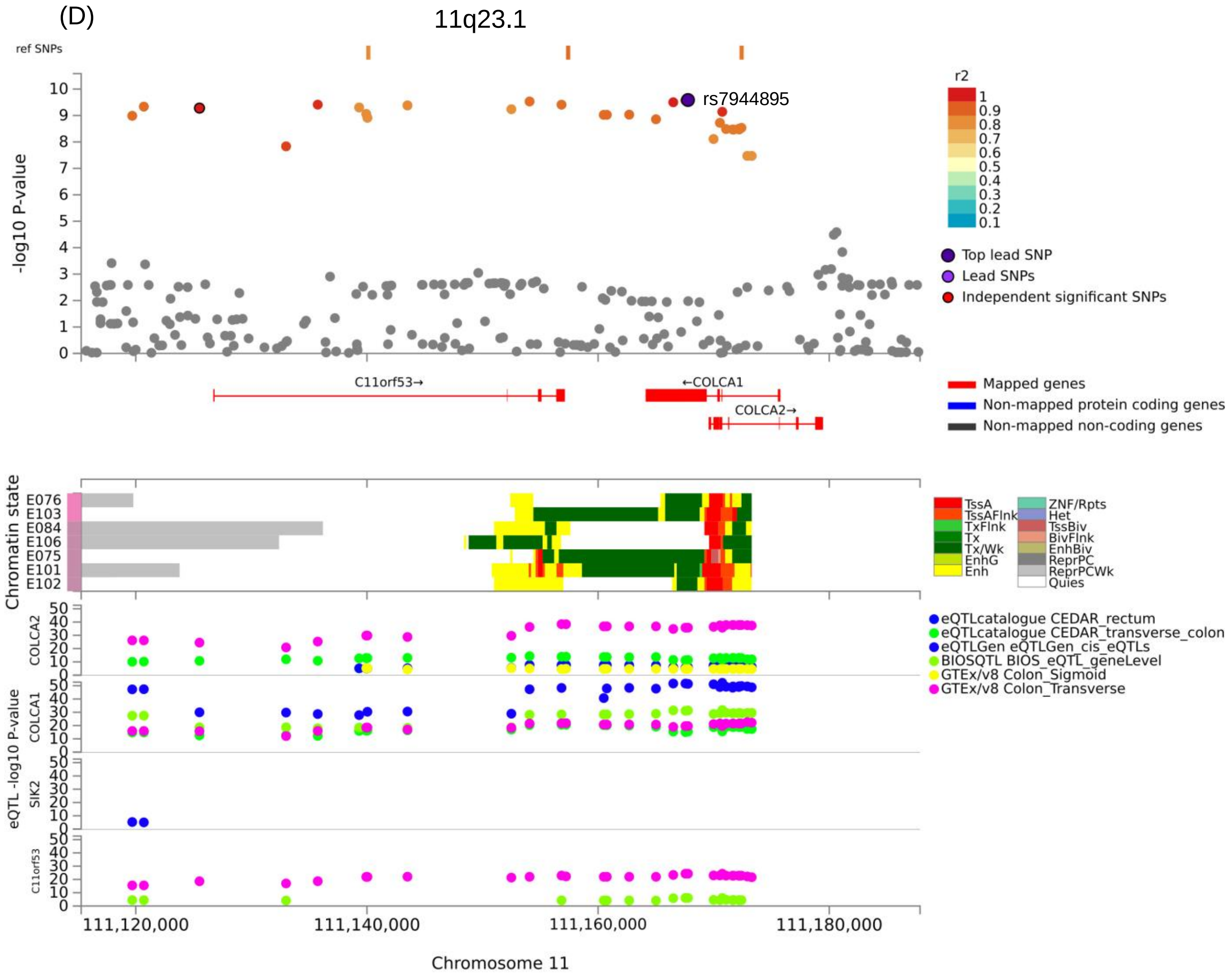
(D)
11q23.1
ref SNPs
rs7944895
-log10 P-value
r2
Top lead SNP
Lead SNPs
Independent significant SNPs
C11orf53→
←COLCA1
COLCA2→
Mapped genes
Non-mapped protein coding genes
Non-mapped non-coding genes
Chromatin state
E076
E103
E084
E106
E075
E101
E102
TssA
TssAFlnk
TxFlnk
Tx
Tx/Wk
EnhG
Enh
ZNF/Rpts
Het
TssBiv
BivFlnk
EnhBiv
ReprPC
ReprPCWk
Quies
COLCA2
COLCA1
SIK2
C11orf53
eQTL -log10 P-value
eQTLcatalogue CEDAR_rectum
eQTLcatalogue CEDAR_transverse_colon
eQTLGen eQTLGen_cis_eQTLs
BIOSQTL BIOS_eQTL_geneLevel
GTEx/v8 Colon_Sigmoid
GTEx/v8 Colon_Transverse
111,120,000
111,140,000
111,160,000
111,180,000
Chromosome 11

(E)

12q13.12

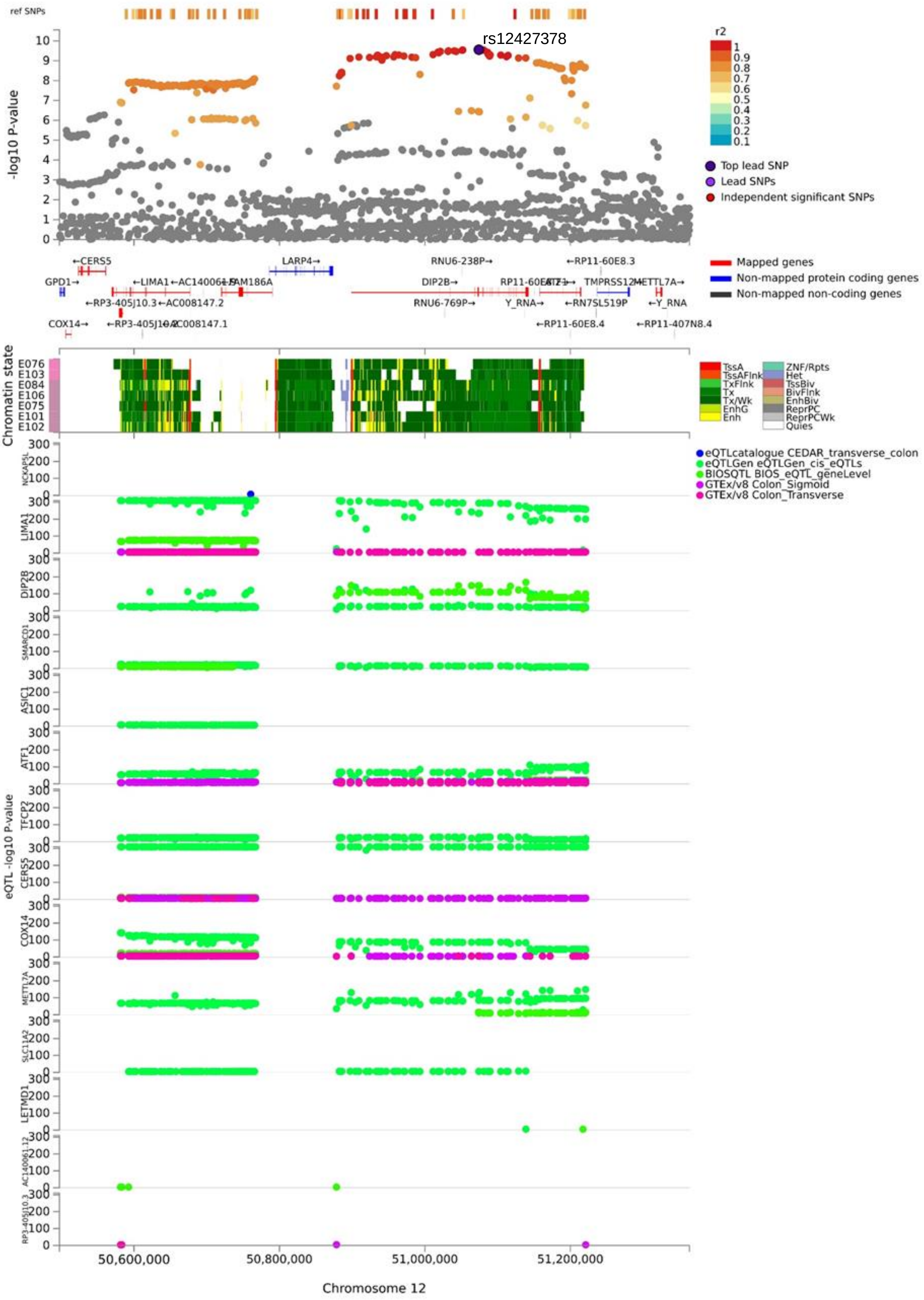

ref SNPs
rs12427378
-log10 P-value
r2
Top lead SNP
Lead SNPs
Independent significant SNPs
Mapped genes
Non-mapped protein coding genes
Non-mapped non-coding genes
Chromatin state
E076
E103
E084
E106
E075
E101
E102
TssA
TssAFlnk
TxFlnk
Tx
Tx/Wk
EnhG
Enh
ZNF/Rpts
Het
TssBiv
BivFlnk
EnhBiv
ReprPC
ReprPCWk
Quies
eQTLcatalogue CEDAR_transverse_colon
eQTLGen eQTLGen_cis_eQTLs
BIOSQTL BIOS_eQTL_geneLevel
GTEx/v8 Colon_Sigmoid
GTEx/v8 Colon_Transverse
eQTL -log10 P-value
LIMA1
DIP2B
SMARCD1
ASIC1
ATF1
TFCP2
CERS5
COX14
METTL7A
SLC11A2
LETMD1
50,600,000
50,800,000
51,000,000
51,200,000
Chromosome 12

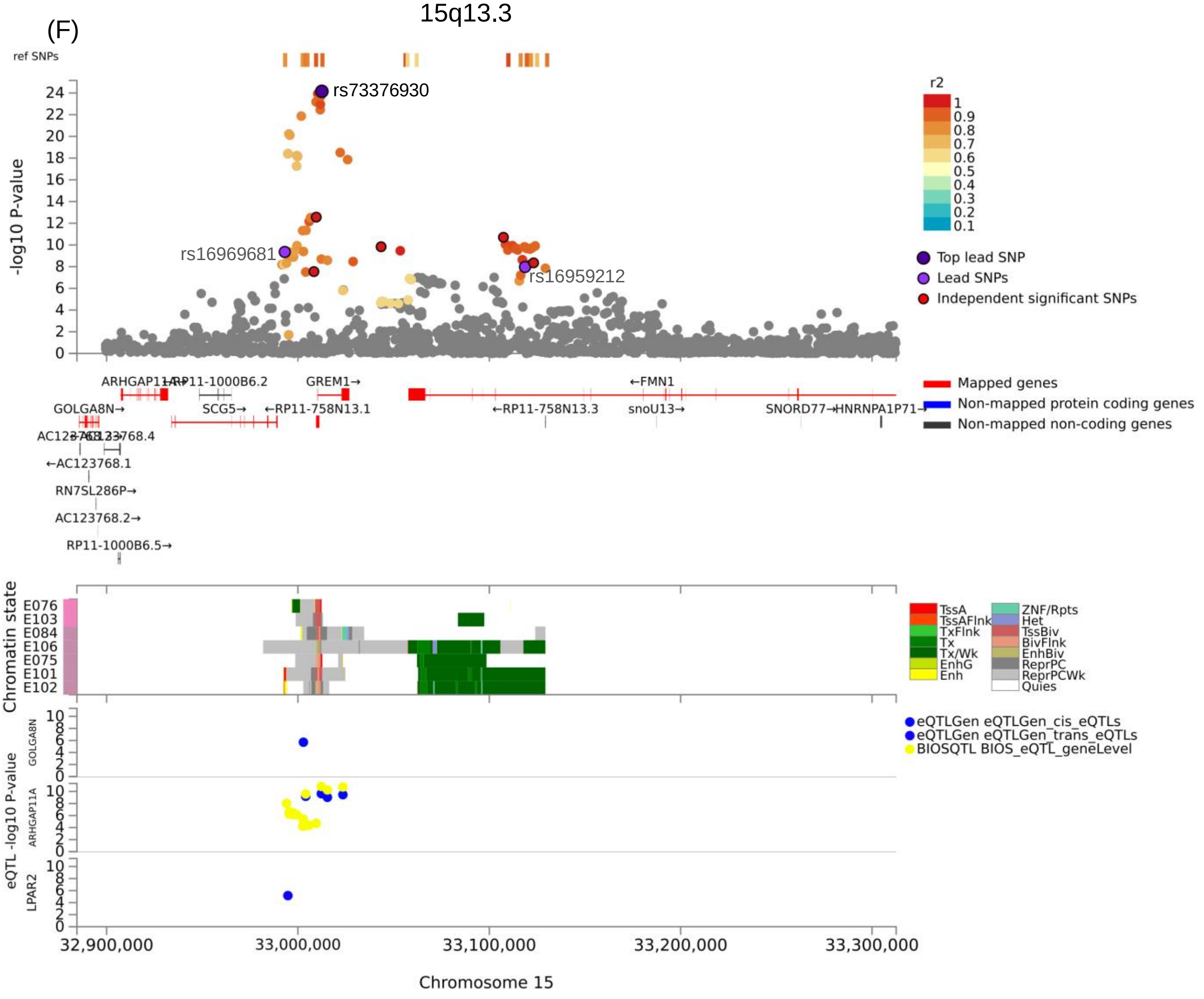
(F)
15q13.3
ref SNPs
rs73376930
rs16969681
rs16959212
-log10 P-value
r2
Top lead SNP
Lead SNPs
Independent significant SNPs
ARHGAP11A
RP11-1000B6.2
GREM1→
←FMN1
GOLGA8N→
SCG5→
←RP11-758N13.1
←RP11-758N13.3
snoU13→
SNORD77→
HNRNPA1P71→
←AC123768.1
RN7SL286P→
AC123768.2→
RP11-1000B6.5→
Mapped genes
Non-mapped protein coding genes
Non-mapped non-coding genes
Chromatin state
E076
E103
E084
E106
E075
E101
E102
TssA
TssAFlnk
TxFlnk
Tx
Tx/Wk
EnhG
Enh
ZNF/Rpts
Het
TssBiv
BivFlnk
EnhBiv
ReprPC
ReprPCWk
Quies
eQTL -log10 P-value
GOLGA8N
ARHGAP11A
LPAR2
eQTLGen eQTLGen_cis_eQTLs
eQTLGen eQTLGen_trans_eQTLs
BIOSQTL BIOS_eQTL_geneLevel
32,900,000
33,000,000
33,100,000
33,200,000
33,300,000
Chromosome 15

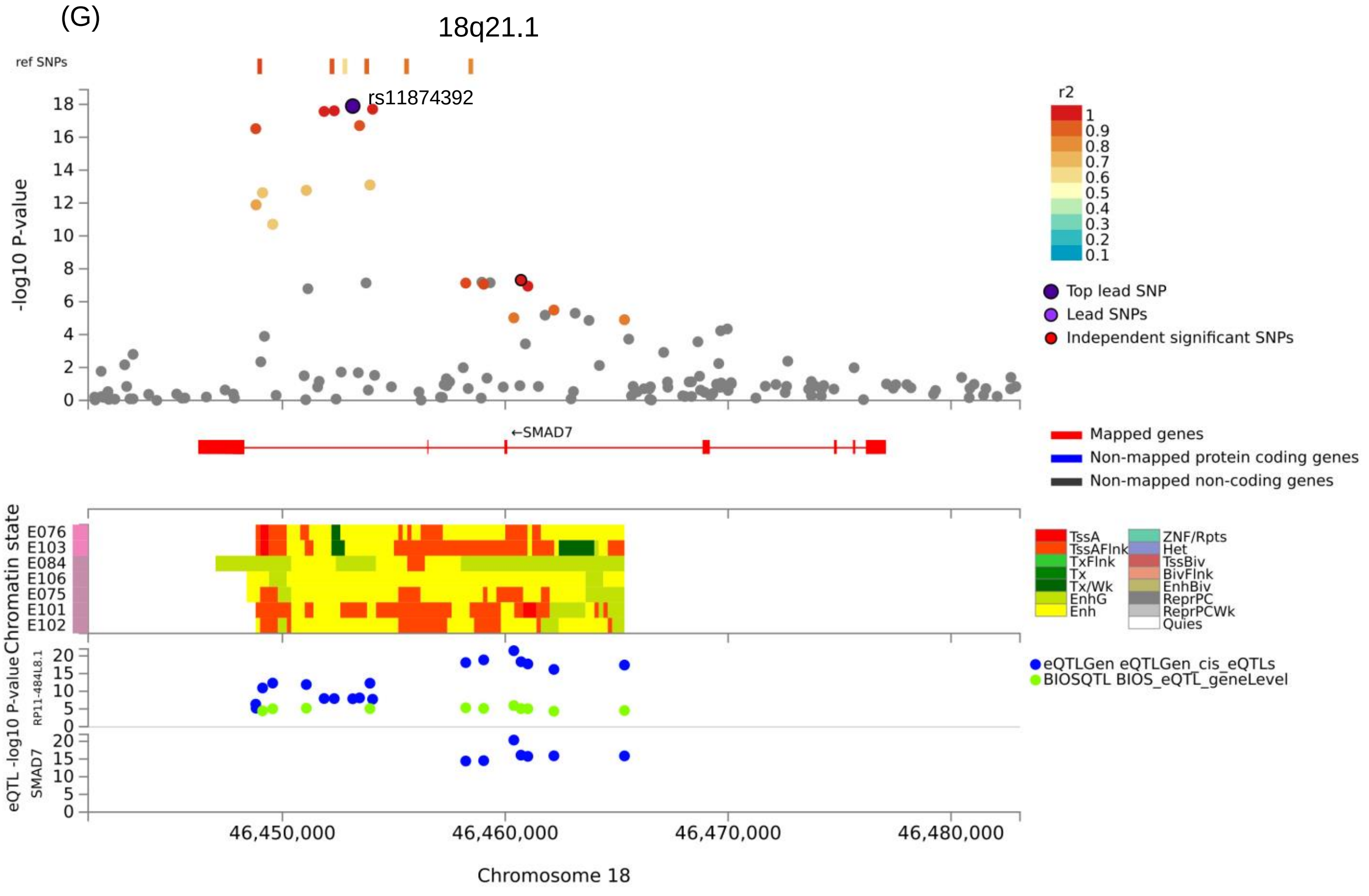
(G)
18q21.1
ref SNPs
rs11874392
-log10 P-value
r2
Top lead SNP
Lead SNPs
Independent significant SNPs
←SMAD7
Mapped genes
Non-mapped protein coding genes
Non-mapped non-coding genes
Chromatin state
E076
E103
E084
E106
E075
E101
E102
TssA
TssAFlnk
TxFlnk
Tx
Tx/Wk
EnhG
Enh
ZNF/Rpts
Het
TssBiv
BivFlnk
EnhBiv
ReprPC
ReprPCWk
Quies
eQTL -log10 P-value
RP11-484L8.1
SMAD7
eQTLGen eQTLGen_cis_eQTLs
BIOSQTL BIOS_eQTL_geneLevel
46,450,000
46,460,000
46,470,000
46,480,000
Chromosome 18

(H) 20p12.3

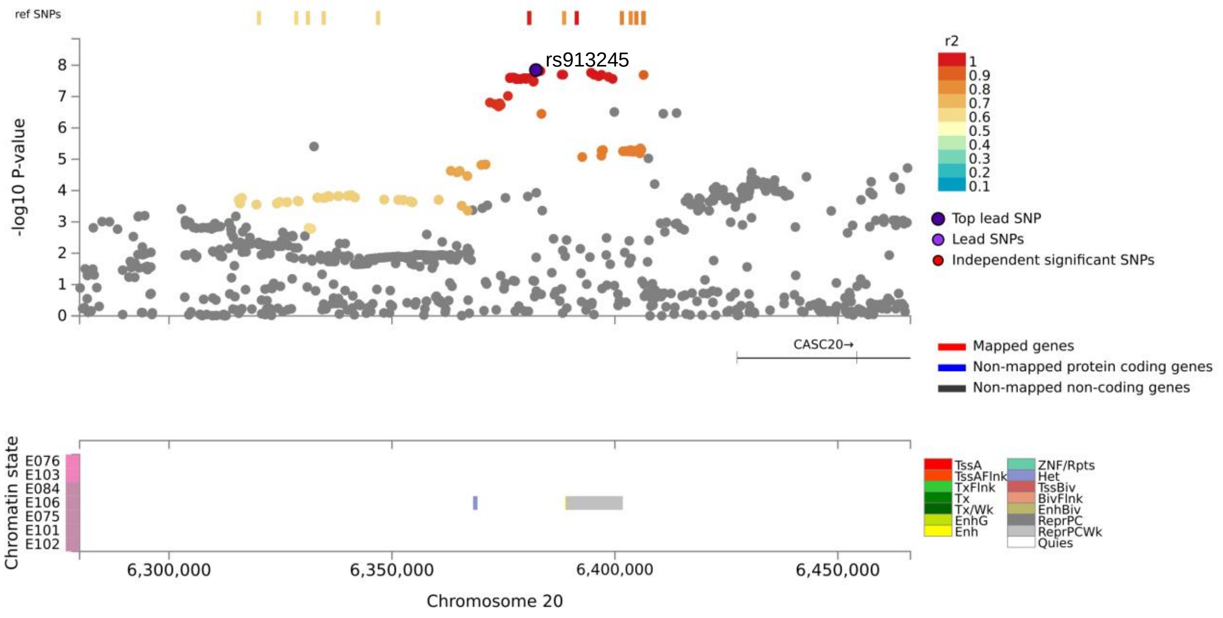

(I) 20q13.13

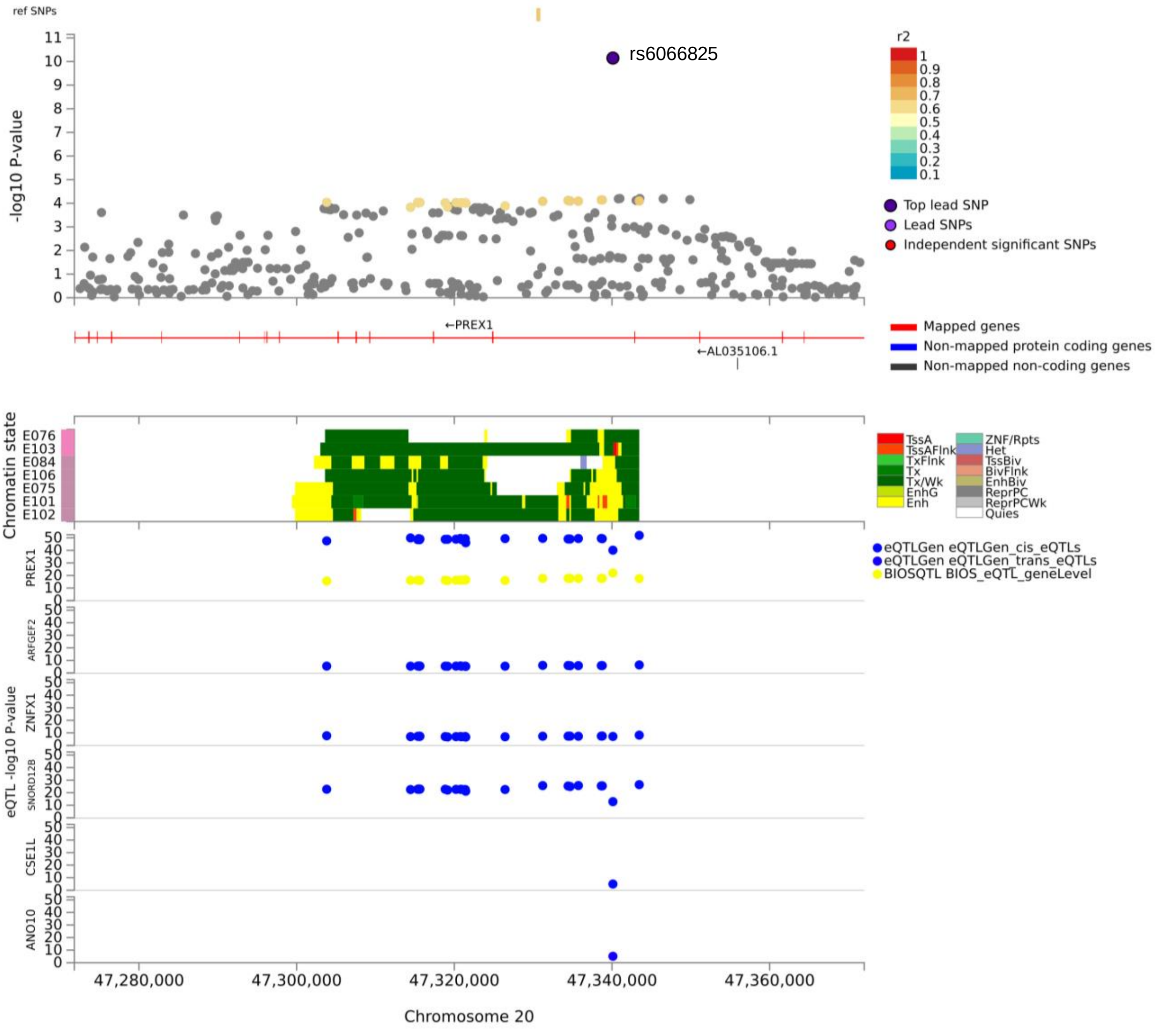

(J)

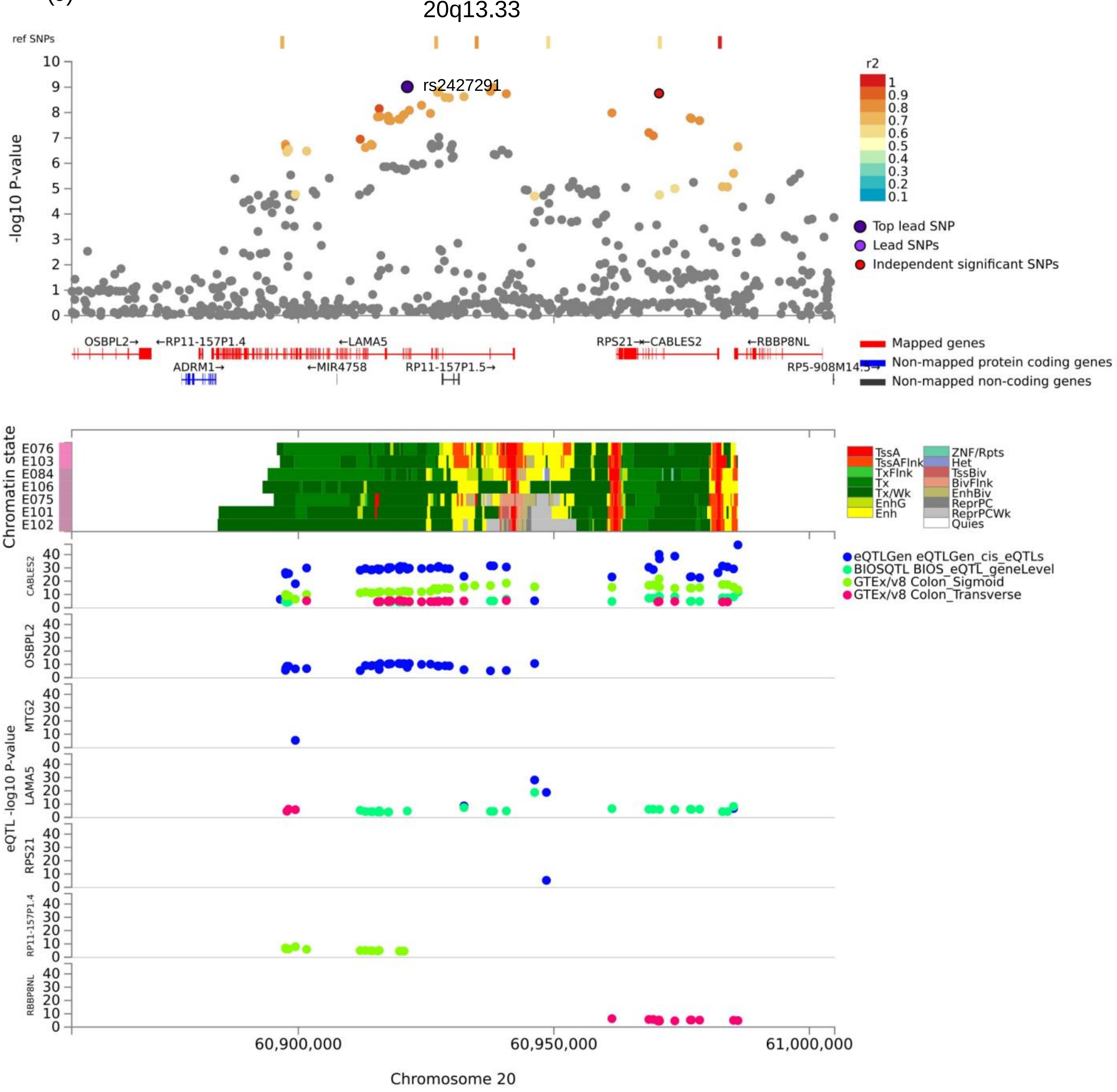

20q13.33
ref SNPs
-log10 P-value
rs2427291
r2
Top lead SNP
Lead SNPs
Independent significant SNPs
OSBPL2→
←RP11-157P1.4
←LAMA5
RPS21→←CABLES2
←RBBP8NL
ADRM1→
←MIR4758
RP11-157P1.5→
RP5-908M14.5→
Mapped genes
Non-mapped protein coding genes
Non-mapped non-coding genes
Chromatin state
E076
E103
E084
E106
E075
E101
E102
TssA
TssAFlnk
TxFlnk
Tx
Tx/Wk
EnhG
Enh
ZNF/Rpts
Het
TssBiv
BivFlnk
EnhBiv
ReprPC
ReprPCWk
Quies
eQTLGen eQTLGen_cis_eQTLs
BIOSQTL BIOS_eQTL_geneLevel
GTEx/v8 Colon_Sigmoid
GTEx/v8 Colon_Transverse
eQTL -log10 P-value
CABLES2
OSBPL2
MTG2
LAMA5
RPS21
RP11-157P1.4
RBBP8NL
60,900,000
60,950,000
61,000,000
Chromosome 20

SUF 4:Enrichment of the credible SNP set for histone marks in different cell-types

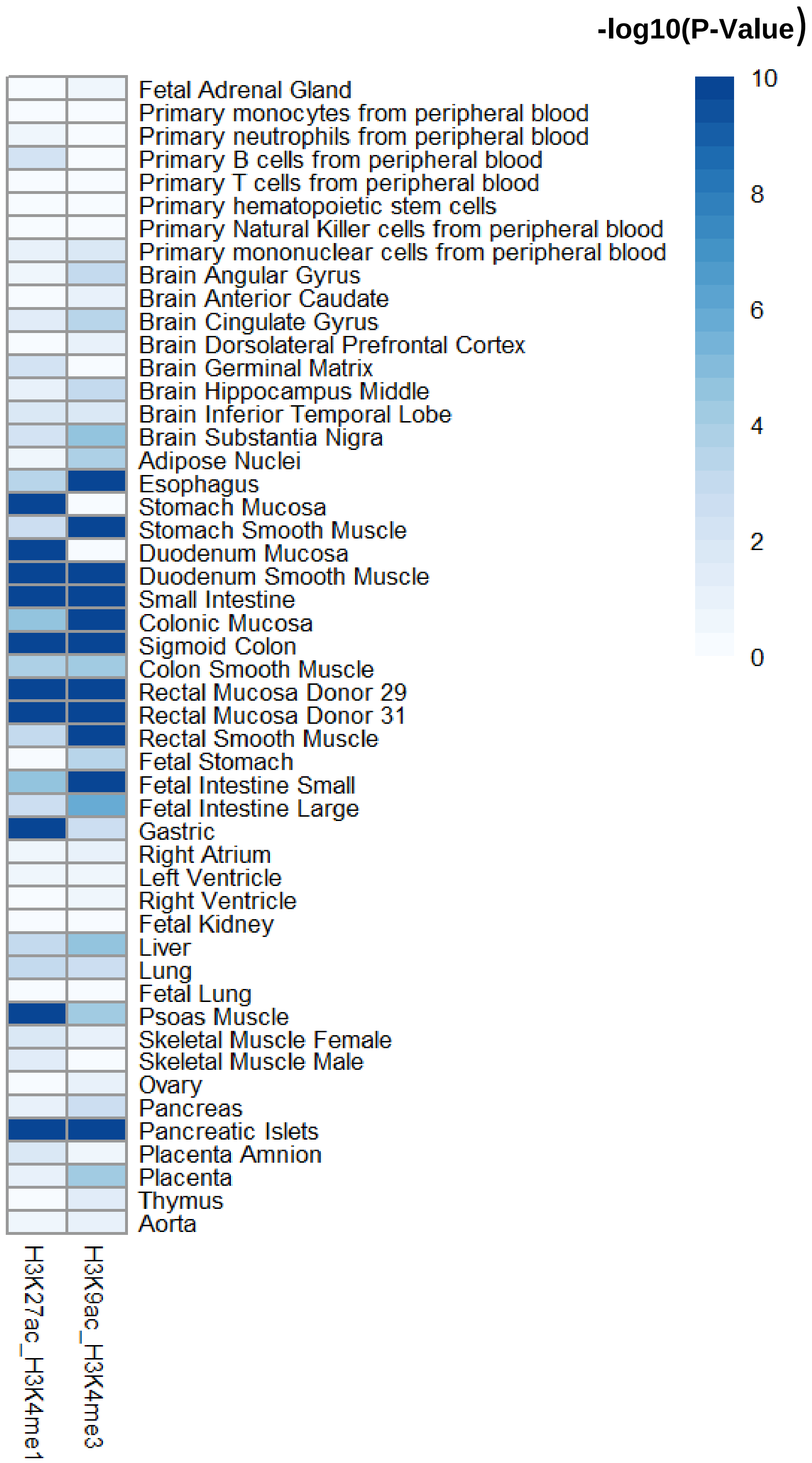

SUF 5A-F: Plots for hi-C chromatin interaction involving the credible SNP set in each loci from the GM12878 cell line. The genes in green represents eQTL mapped genes, orange represents chromatin interaction mapped and red are both eQTL and Chromatin interaction mapped genes at each locus.

(A)

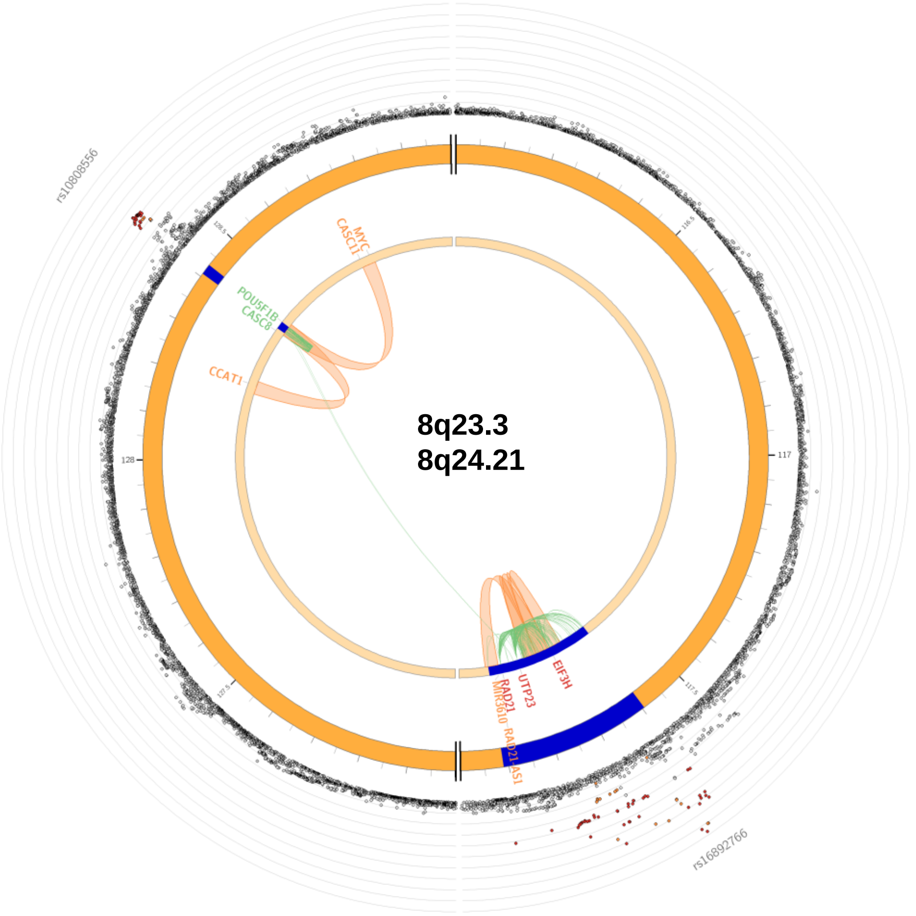

(B)

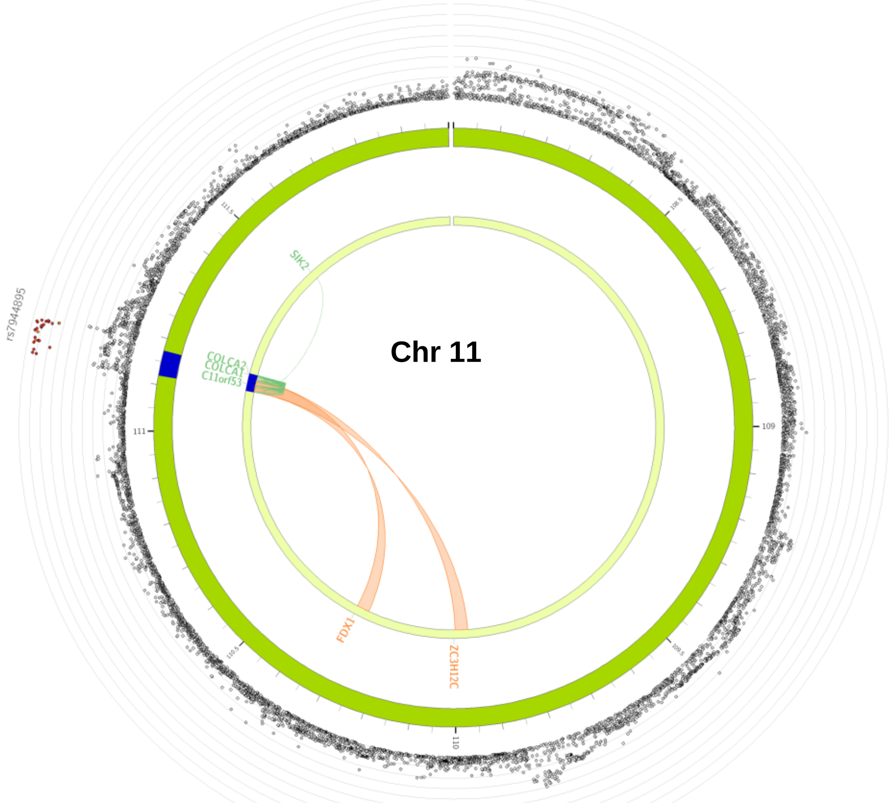

(C)

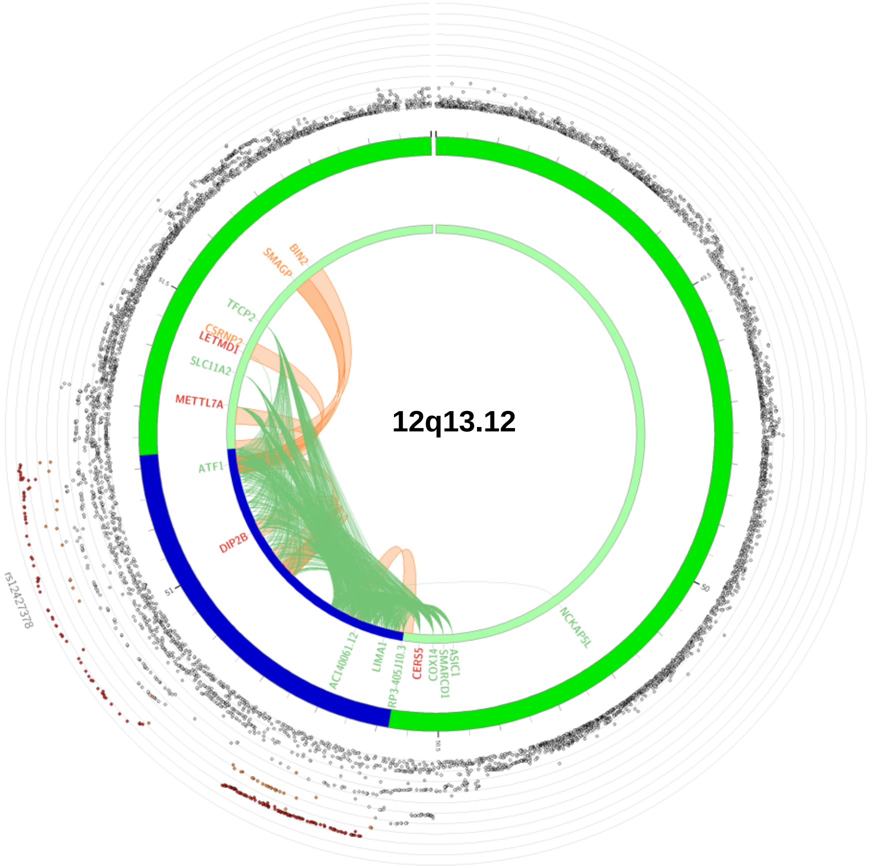

12q13.12
BIN2
SMAGP
TFCP2
CSRNP2
LETMD1
SLC11A2
METTL7A
ATF1
DIP2B
AC140061.12
LIMA1
RP3-405J10.3
CERS5
COX14
SMARCD1
ASIC1
NCKAP5L
rs12427378

(D)

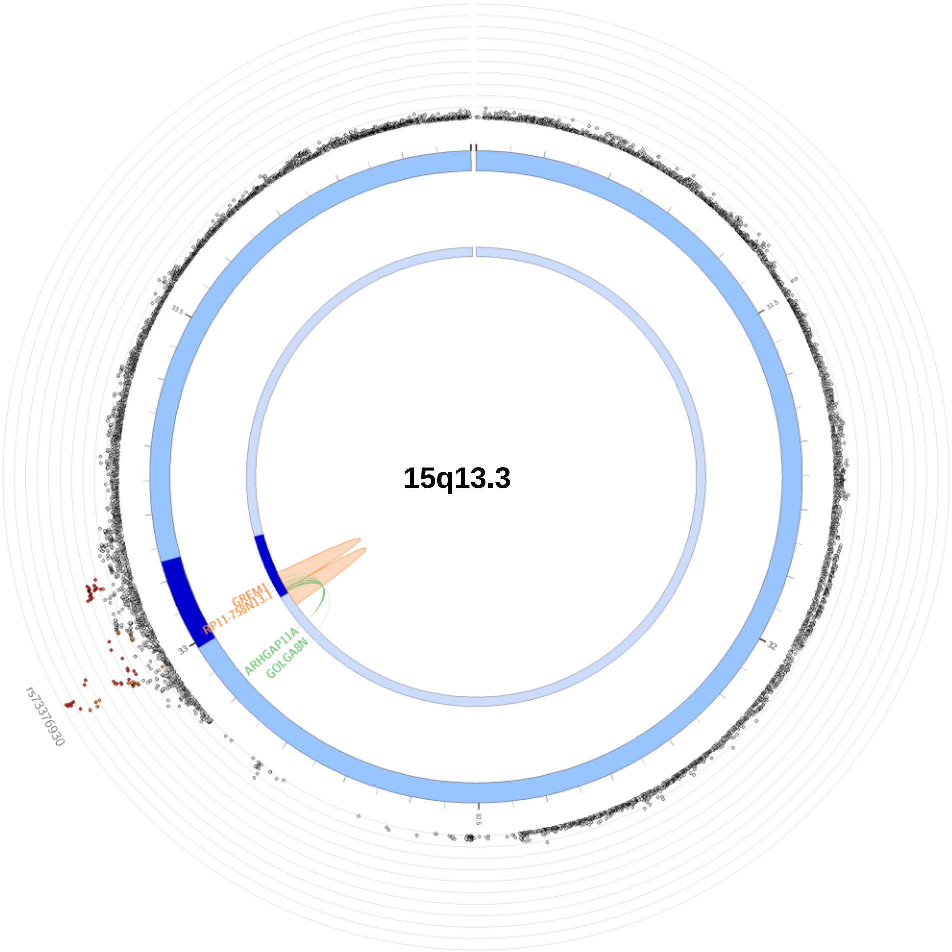

15q13.3
GREM1
RP11-758N13.1
ARHGAP11A
GOLGA8N
rs73376930

(E)

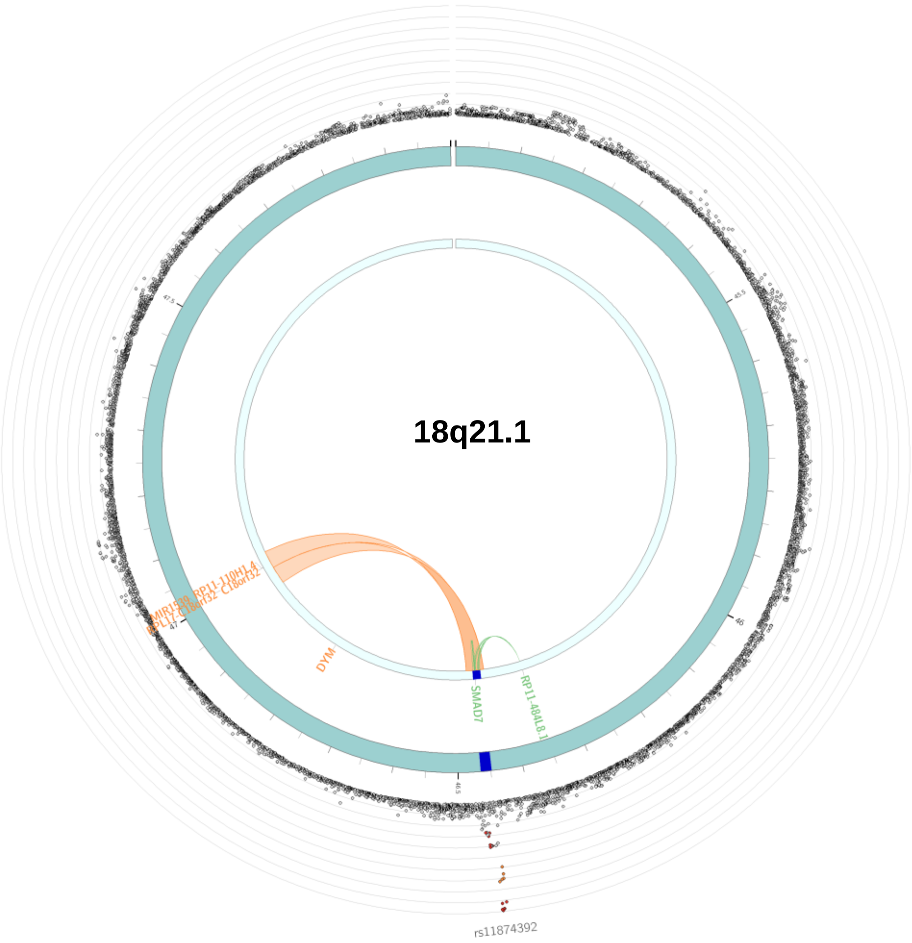
18q21.1
DYM
SMAD7
rs11874392

(F)

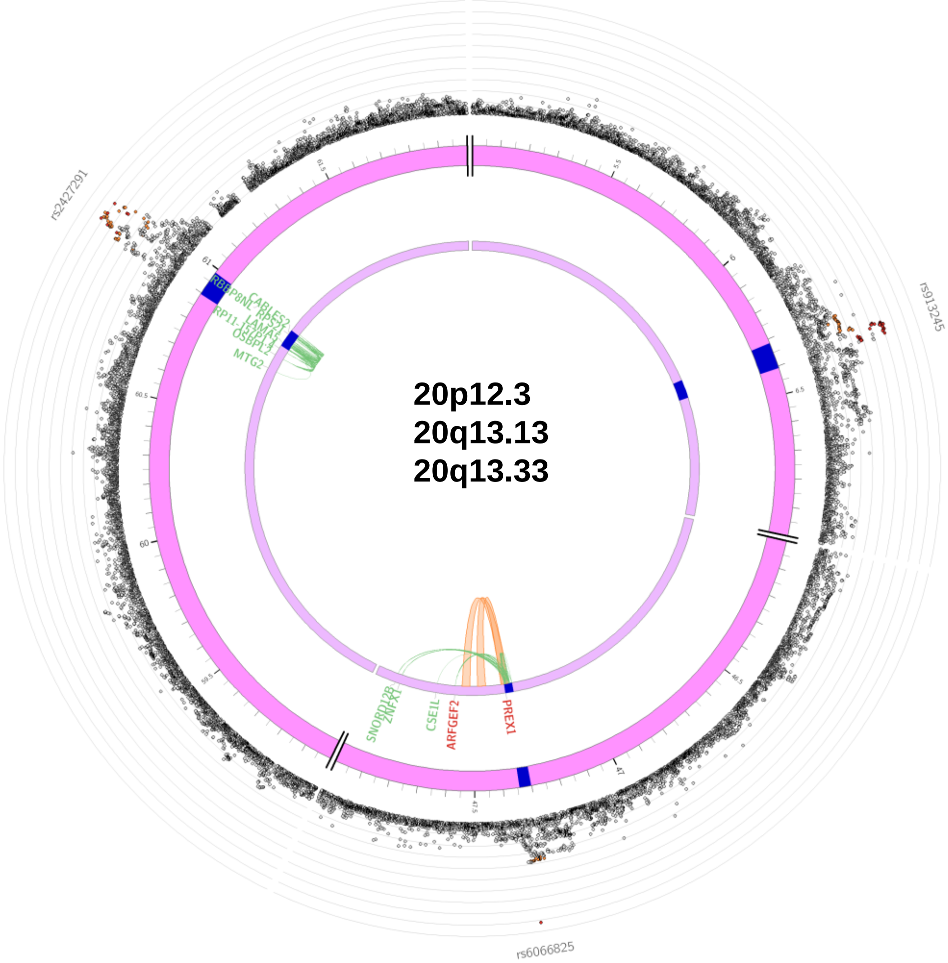
20p12.3
20q13.13
20q13.33
rs2427291
rs913245
MTG2
CSE1L
ARFGEF2
PREX1
rs6066825

~~SUF6A~~: **Gene-level associations:** Gene-level associations with the y-axis representing the P-value (−log10) for association in the MAGMA. The gene-level genome-wide significance threshold in MAGMA was P = 2.6e-06 (after accounting for 19,235 genes tested) indicated by the dashed line.

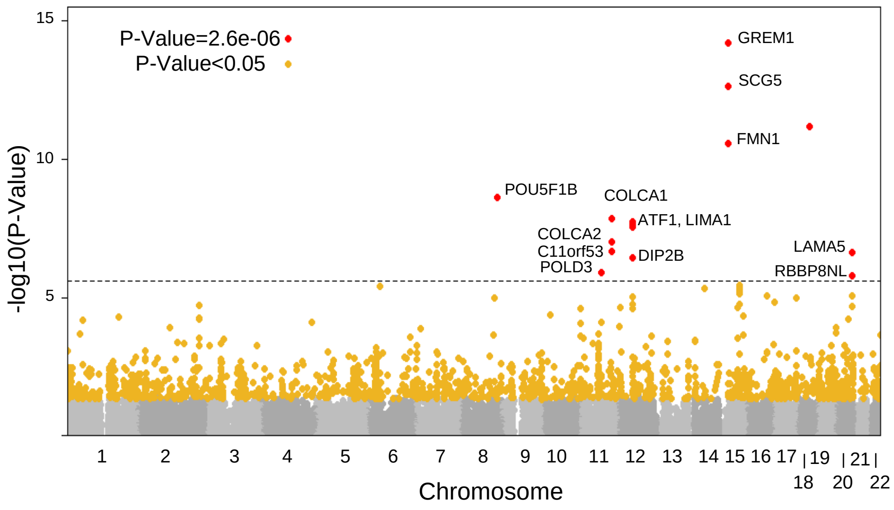

~~SUF6B~~: **Gene-level associations:** The largest protein–protein interaction (PPI) biological subnetwork using proteins encoded by genes with nominal significance (P<0.05) in MAGMA as "seeds". The orange-colored nodes indicate seed proteins that interact with at least two other proteins in this subnetwork with larger nodes representing the seed nodes with higher number of interactions. Seed proteins encircled by red are the proteins encoded by genes identified in the previous CRC GWAS. Green nodes are other non-seed proteins that are in the subnetwork.

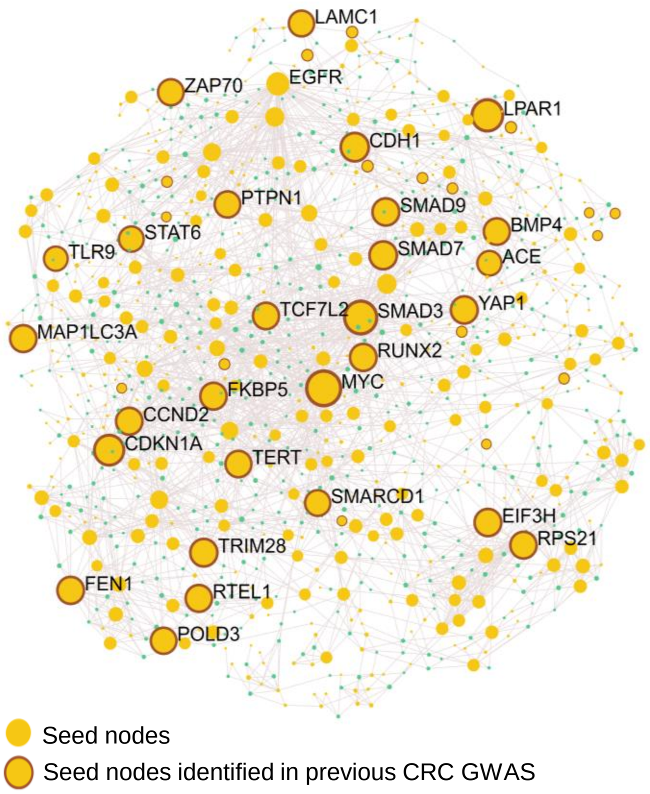

SUF 7: Statistical power calculation to detect OR of 1.8 with 0.8% MAF in a one-stage study

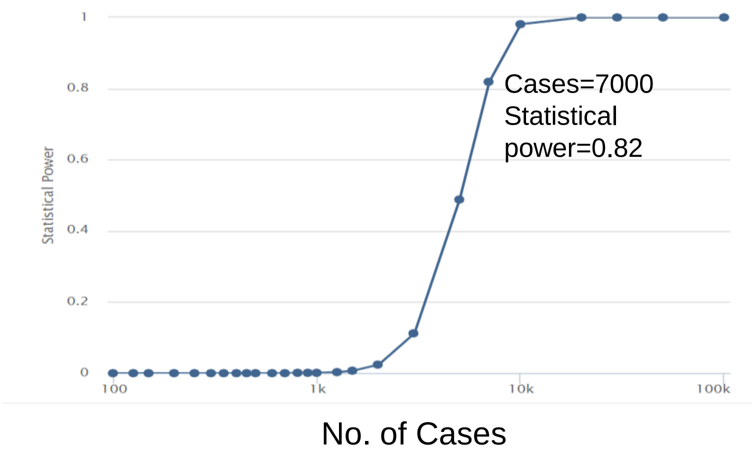

SUF 8: Comparison of the expression of the prioritized genes in colorectal cancer tissues depicted by red colour [T] (COAD=Colon adenocarcinoma, READ= Rectal adenocarcinoma) and normal colonic cells in green colour [N] from TCGA and GTEx datasets respectively. * P<0.05

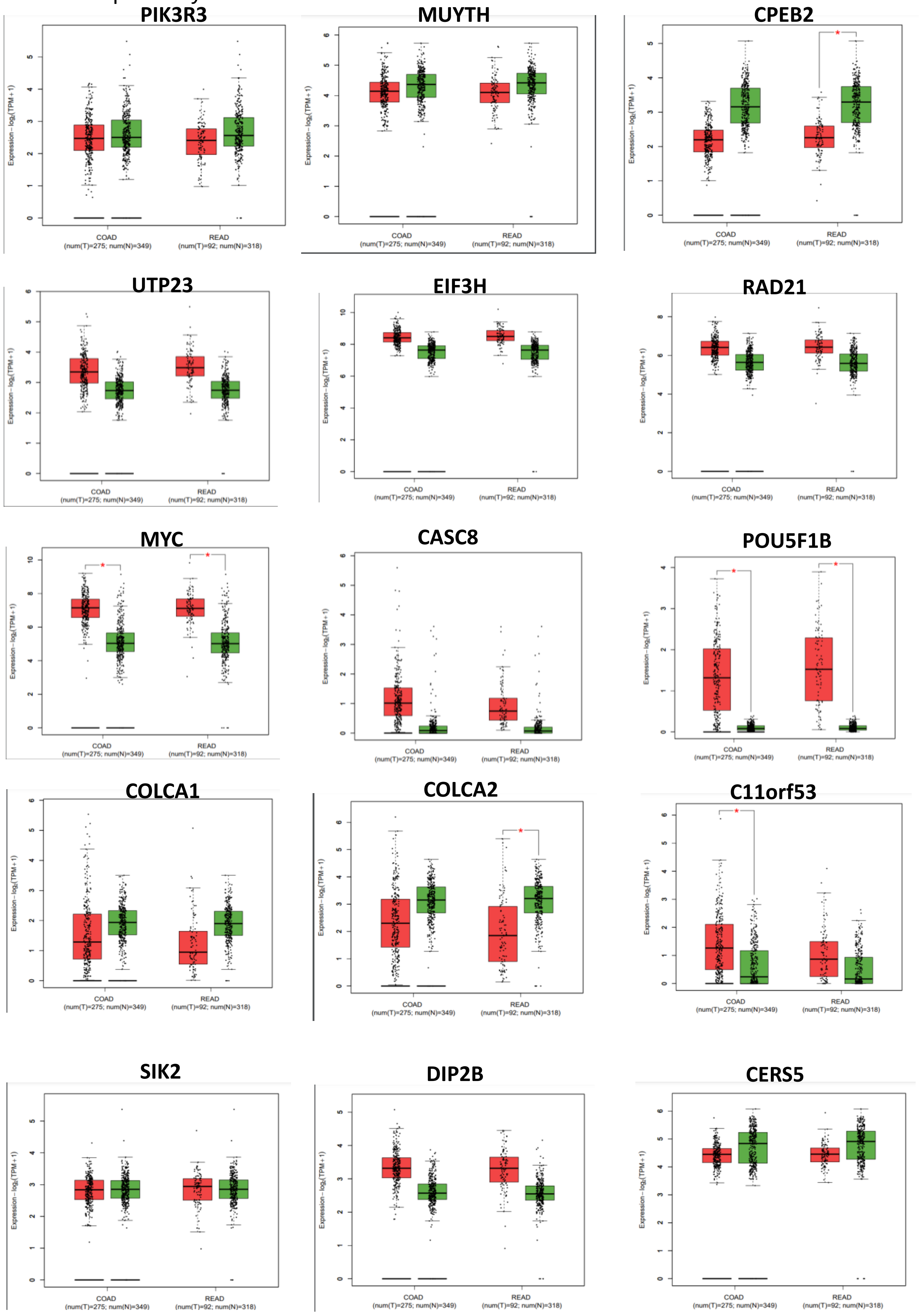

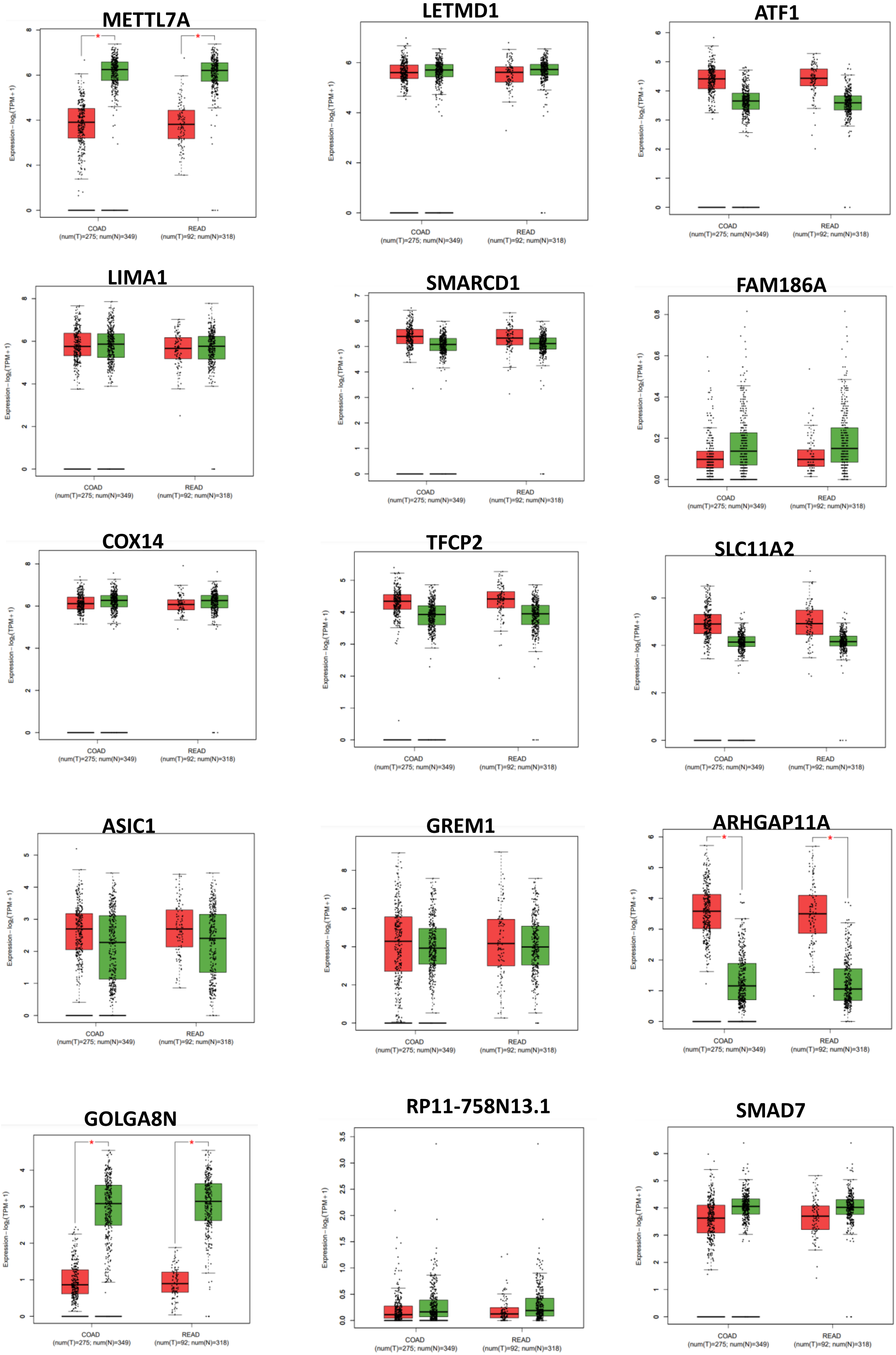
METTL7A
LETMD1
ATF1
LIMA1
SMARCD1
FAM186A
COX14
TFCP2
SLC11A2
ASIC1
GREM1
ARHGAP11A
GOLGA8N
RP11-758N13.1
SMAD7
Expression – log2(TPM + 1)
COAD
(num(T)=275; num(N)=349)
READ
(num(T)=92; num(N)=318)

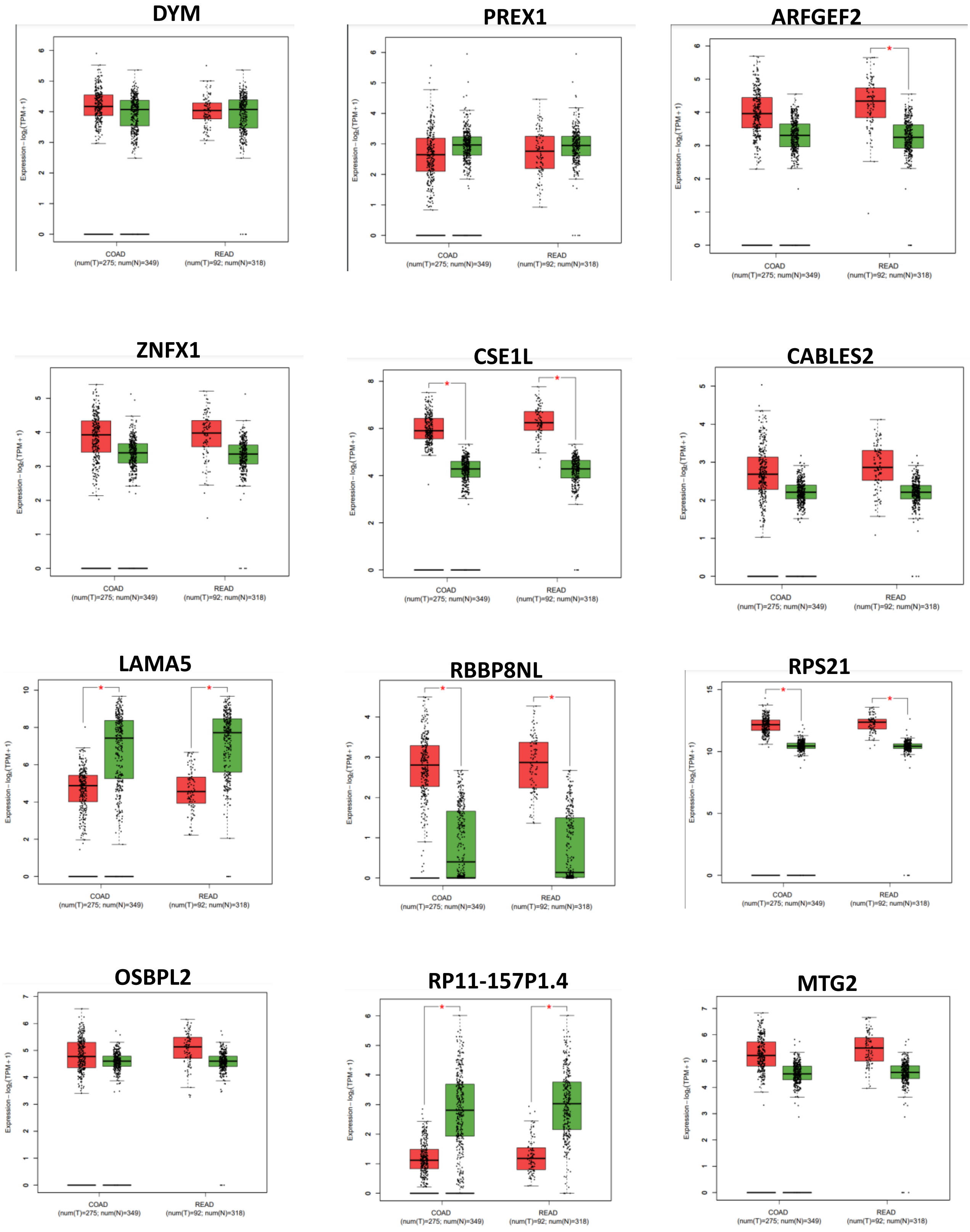
DYM
PREX1
ARFGEF2
ZNFX1
CSE1L
CABLES2
LAMA5
RBBP8NL
RPS21
OSBPL2
RP11-157P1.4
MTG2
Expression − log2(TPM + 1)
COAD
(num(T)=275; num(N)=349)
READ
(num(T)=92; num(N)=318)

~~SUF 9~~: Scatter plot of the beta estimates from exposure and outcome GWAS for the tested risk factors

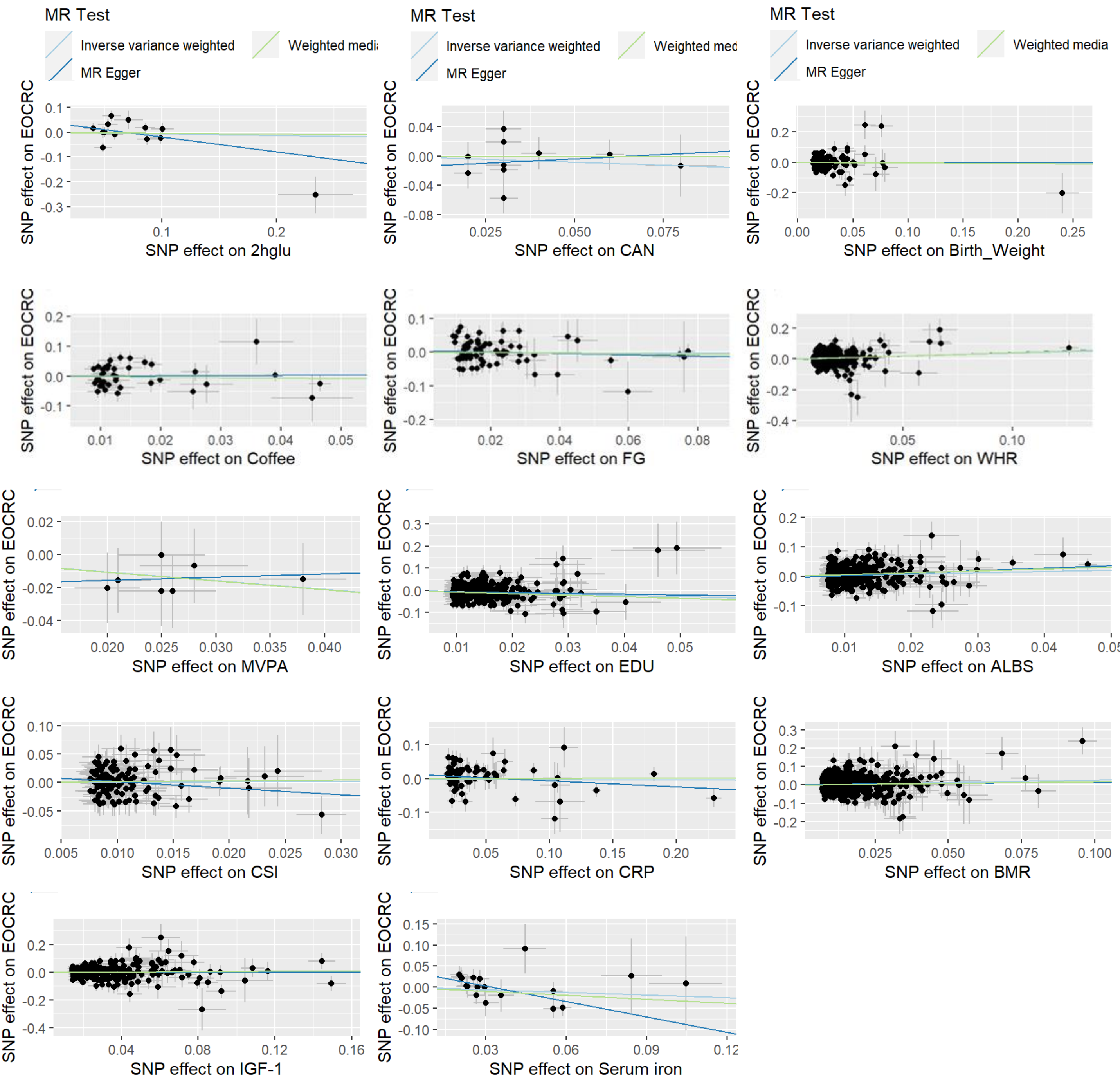

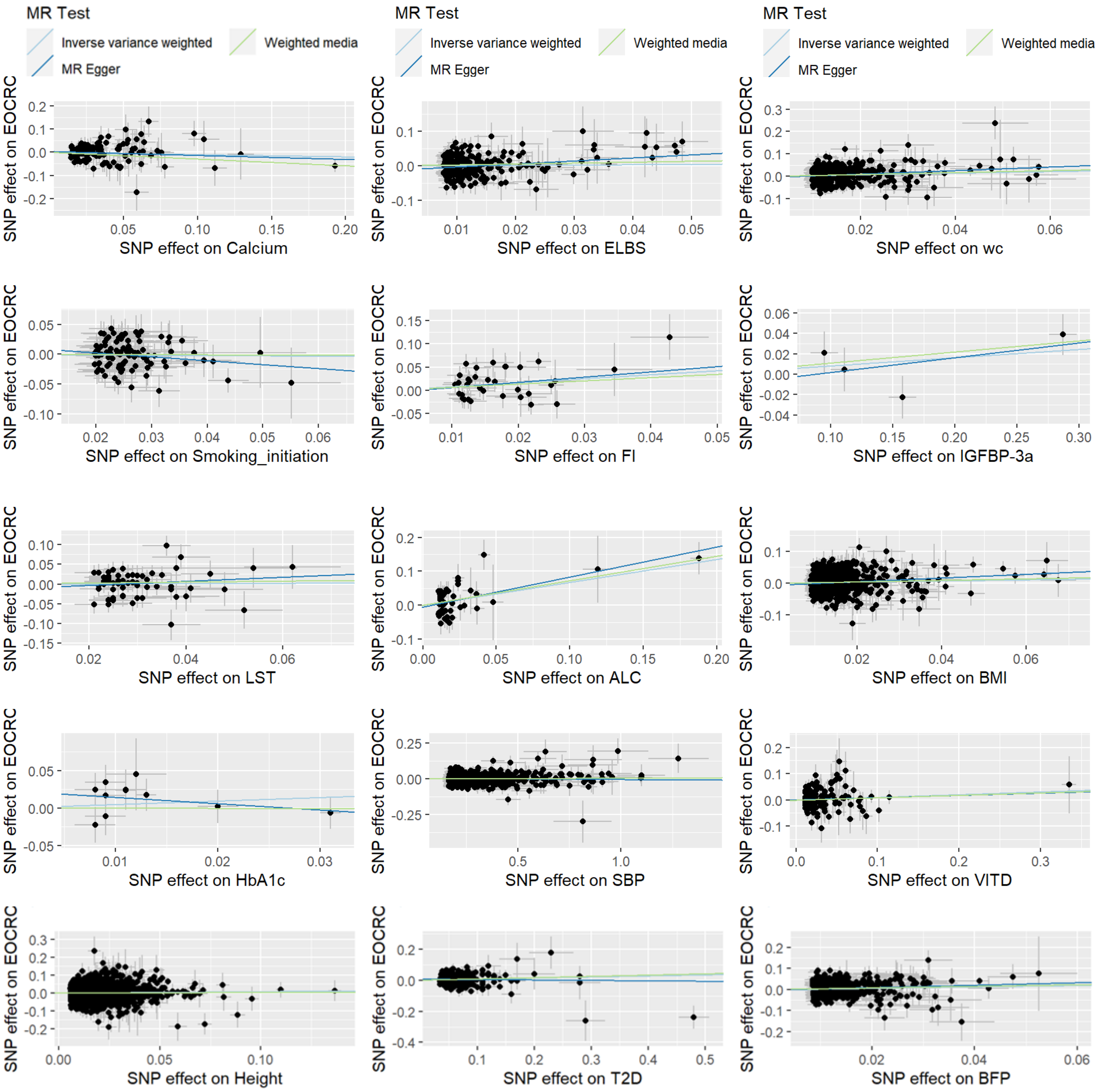
MR Test
Inverse variance weighted
Weighted media
MR Egger
SNP effect on EOCRC
SNP effect on Calcium
SNP effect on ELBS
SNP effect on wc
SNP effect on Smoking_initiation
SNP effect on FI
SNP effect on IGFBP-3a
SNP effect on LST
SNP effect on ALC
SNP effect on BMI
SNP effect on HbA1c
SNP effect on SBP
SNP effect on VITD
SNP effect on Height
SNP effect on T2D
SNP effect on BFP

~~SUF 10~~: Odds ratios from inverse variance weighted MR-analysis for association between putative risk factors and overall CRC

Exposure	nSNPs	P-value	OR	95%-CI
**Body size**				
Waist to hip ratio	481	1.62e-09	1.24	[1.16; 1.33]
Waist circumference	475	4.16e-12	1.25	[1.18; 1.34]
Body fat percentage	548	1.46e-06	1.20	[1.12; 1.30]
Basal metabolic rate	974	3.41e-05	1.15	[1.08; 1.23]
Body mass index	826	2.44e-06	1.12	[1.07; 1.18]
Early life body size	235	0.76	1.02	[0.91; 1.15]
Birth weight	163	0.02	1.13	[1.02; 1.26]
Height	3373	0.02	1.03	[1.01; 1.06]
**Diet and lifestyle**				
Alcoholic drinks per week	48	0.11	1.14	[0.97; 1.34]
Lifetime smoking index	106	0.007	1.19	[1.05; 1.35]
Smoking initiation	85	0.4	1.04	[0.95; 1.14]
25(OH)D	150	0.59	1.02	[0.95; 1.09]
Coffee consumption	38	0.17	1.18	[0.93; 1.51]
Systolic blood pressure	649	0.03	0.92	[0.85; 0.99]
Serum calcium	104	0.28	1.00	[1.00; 1.00]
Serum iron	18	0.99	1.00	[0.82; 1.22]
Leisure screen time	59	0.88	1.01	[0.92; 1.10]
Physical activity (MVPA)	7	0.003	0.80	[0.69; 0.93]
Educational attaintment	412	1.15e-11	0.32	[0.23; 0.45]
**Glycemic traits**				
Fasting insulin	33	0.001	1.78	[1.26; 2.52]
HbA1c	9	0.61	0.95	[0.78; 1.15]
Type 2 diabetes	212	0.41	1.01	[0.98; 1.04]
Fasting glucose	58	0.37	1.14	[0.85; 1.53]
2hour glucose	11	0.91	0.99	[0.89; 1.11]
**Inflammation and growth factors**				
C-reactive protein	65	0.44	0.96	[0.88; 1.06]
Circulating adeponectin	9	0.18	0.94	[0.85; 1.03]
IGFBP3	2	1.37e-08	1.09	[1.06; 1.13]
IGF1	217	0.004	1.02	[1.01; 1.04]

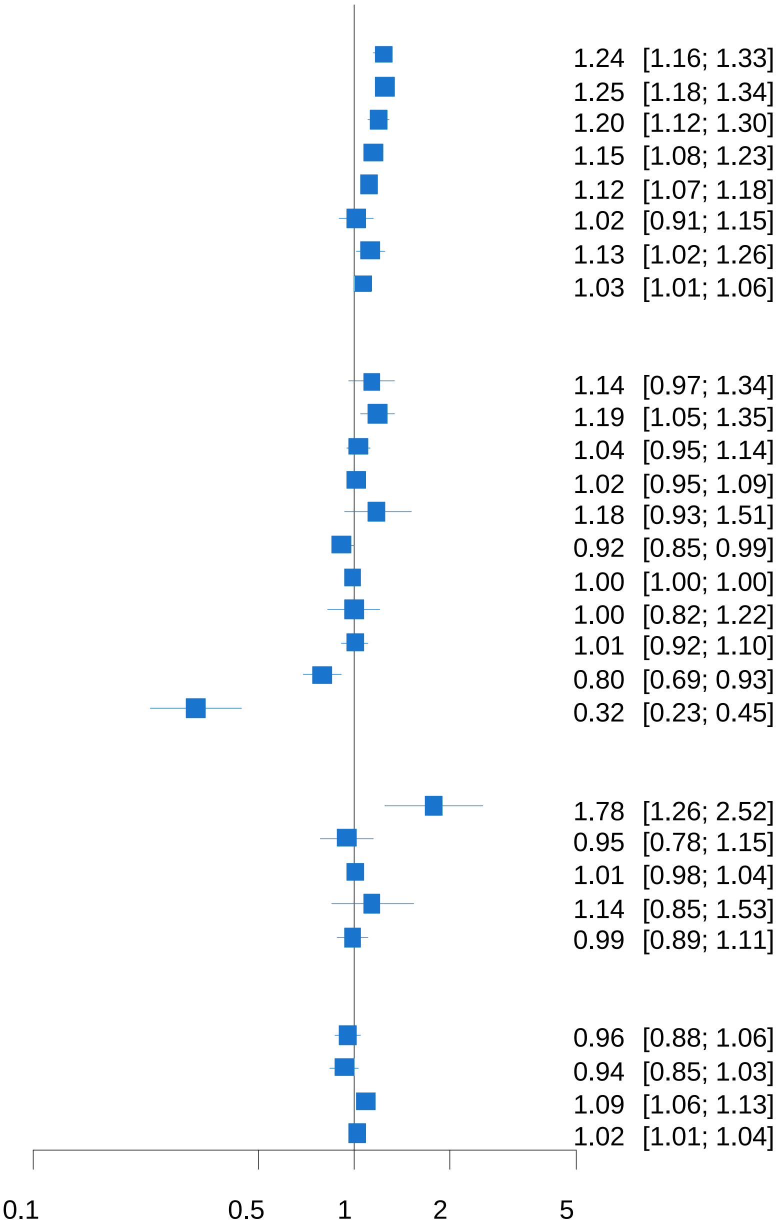

All associations are expressed as OR per SD increase in the risk factor except for alcoholic drinks per week and fasting insulin which were expressed as OR per unit increase in natural logarithm of the exposures. For categorical risk factors like smoking initiation (ever vs never), type 2 diabetes (yes vs no) and physical activity (inactive vs active), the ORs were expressed as unit change in the exposure, compared to the reference group

Funding:
Genetics and Epidemiology of Colorectal Cancer Consortium (GECCO): National Cancer Institute, National Institutes of Health, U.S. Department of Health and Human Services (U01 CA137088, R01 CA059045, R01 201407). Genotyping/Sequencing services were provided by the Center for Inherited Disease Research (CIDR) contract number HHSN268201700006I and HHSN268201200008I . This research was funded in part through the NIH/NCI Cancer Center Support Grant P30 CA015704. Scientific Computing Infrastructure at Fred Hutch funded by ORIP grant S10OD028685.
ASTERISK: a Hospital Clinical Research Program (PHRC-BRD09/C) from the University Hospital Center of Nantes (CHU de Nantes) and supported by the Regional Council of Pays de la Loire, the Groupement des Entreprises Françaises dans la Lutte contre le Cancer (GEFLUC), the Association Anne de Bretagne Génétique and the Ligue Régionale Contre le Cancer (LRCC).
The ATBC Study is supported by the Intramural Research Program of the U.S. National Cancer Institute, National Institutes of Health, Department of Health and Human Services.
CLUE II funding was from the National Cancer Institute (U01 CA086308, Early Detection Research Network; P30 CA006973), National Institute on Aging (U01 AG018033), and the American Institute for Cancer Research. The content of this publication does not necessarily reflect the views or policies of the Department of Health and Human Services, nor does mention of trade names, commercial products, or organizations imply endorsement by the US government.
Maryland Cancer Registry (MCR)
Cancer data was provided by the Maryland Cancer Registry, Center for Cancer Prevention and Control, Maryland Department of Health, with funding from the State of Maryland and the Maryland Cigarette Restitution Fund. The collection and availability of cancer registry data is also supported by the Cooperative Agreement NU58DP006333, funded by the Centers for Disease Control and Prevention. Its contents are solely the responsibility of the authors and do not necessarily represent the official views of the Centers for Disease Control and Prevention or the Department of Health and Human Services.
ColoCare: This work was supported by the National Institutes of Health (grant numbers R01 CA189184 (Li/Ulrich), U01 CA206110 (Ulrich/Li/Siegel/Figueiredo/Colditz, 2P30CA015704- 40 (Gilliland), R01 CA207371 (Ulrich/Li)), the Matthias Lackas-Foundation, the German Consortium for Translational Cancer Research, and the EU TRANSCAN initiative.
The Colon Cancer Family Registry (CCFR, www.coloncfr.org) is supported in part by funding from the National Cancer Institute (NCI), National Institutes of Health (NIH) (award U01 CA167551). Support for case ascertainment was provided in part from the Surveillance, Epidemiology, and End Results (SEER) Program and the following U.S. state cancer registries: AZ, CO, MN, NC, NH; and by the Victoria Cancer Registry (Australia) and Ontario Cancer Registry (Canada). The CCFR Set-1 (Illumina 1M/1M-Duo) and Set-2 (Illumina Omni1-Quad) scans were supported by NIH awards U01 CA122839 and R01 CA143237 (to GC). The CCFR Set-3 (Affymetrix Axiom CORECT Set array) was supported by NIH award U19 CA148107 and R01 CA81488 (to SBG). The CCFR Set-4 (Illumina OncoArray 600K SNP array) was supported by NIH award U19 CA148107 (to SBG) and by the Center for Inherited Disease Research (CIDR), which is funded by the NIH to the Johns Hopkins University, contract number HHSN268201200008I. Additional funding for the OFCCR/ARCTIC was through award GL201-043 from the Ontario Research Fund (to BWZ), award 112746 from the Canadian Institutes of Health Research (to TJH), through a Cancer Risk Evaluation (CaRE) Program grant from the Canadian Cancer Society (to SG), and through generous support from the Ontario Ministry of Research and Innovation. The SFCCR Illumina HumanCytoSNP array was supported in part through NCI/NIH awards U01/U24 CA074794 and R01 CA076366 (to PAN). The content of this manuscript does not necessarily reflect the views or policies of the NCI, NIH or any of the collaborating centers in the Colon Cancer Family Registry (CCFR), nor does mention of trade names, commercial products, or organizations imply endorsement by the US Government, any cancer registry, or the CCFR.

COLON: The COLON study is sponsored by Wereld Kanker Onderzoek Fonds, including funds from grant 2014/1179 as part of the World Cancer Research Fund International Regular Grant Programme, by Alpe d'Huzes and the Dutch Cancer Society (UM 2012–5653, UW 2013-5927, UW2015-7946), and by TRANSCAN (JTC2012-MetaboCCC, JTC2013-FOCUS). The Nqplus study is sponsored by a ZonMW investment grant (98-10030); by PREVIEW, the project PREVention of diabetes through lifestyle intervention and population studies in Europe and around the World (PREVIEW) project which received funding from the European Union Seventh Framework Programme (FP7/2007–2013) under grant no. 312057; by funds from TI Food and Nutrition (cardiovascular health theme), a public–private partnership on precompetitive research in food and nutrition; and by FOODBALL, the Food Biomarker Alliance, a project from JPI Healthy Diet for a Healthy Life.
COLO2&3: National Institutes of Health (R01 CA060987).
Colorectal Cancer Transdisciplinary (CORECT) Study: The CORECT Study was supported by the National Cancer Institute, National Institutes of Health (NCI/NIH), U.S. Department of Health and Human Services (grant numbers U19 CA148107, R01 CA081488, P30 CA014089, R01 CA197350; P01 CA196569; R01 CA201407; R01 CA242218), National Institutes of Environmental Health Sciences, National Institutes of Health (grant number T32 ES013678) and a generous gift from Daniel and Maryann Fong.
CORSA: The CORSA study was funded by Austrian Research Funding Agency (FFG) BRIDGE (grant 829675, to Andrea Gsur), the "Herzfelder'sche Familienstiftung" (grant to Andrea Gsur) and was supported by COST Action BM1206.
CPS-II: The American Cancer Society funds the creation, maintenance, and updating of the Cancer Prevention Study-II (CPS-II) cohort. The study protocol was approved by the institutional review boards of Emory University, and those of participating registries as required.
CRCGEN: Colorectal Cancer Genetics & Genomics, Spanish study was supported by Instituto de Salud Carlos III, co-funded by FEDER funds –a way to build Europe– (grants PI14-613 and PI09-1286), Agency for Management of University and Research Grants (AGAUR) of the Catalan Government (grant 2017SGR723), Junta de Castilla y León (grant LE22A10-2), the Spanish Association Against Cancer (AECC) Scientific Foundation grant GCTRA18022MORE and the Consortium for Biomedical Research in Epidemiology and Public Health (CIBERESP), action Genrisk. Sample collection of this work was supported by the Xarxa de Bancs de Tumors de Catalunya sponsored by Pla Director d'Oncología de Catalunya (XBTC), Plataforma Biobancos PT13/0010/0013 and ICOBIOBANC, sponsored by the Catalan Institute of Oncology. We thank CERCA Programme, Generalitat de Catalunya for institutional support.
Czech Republic CCS: This work was supported by the Grant Agency of the Czech Republic (21-04607X, 21-27902S), by the Grant Agency of the Ministry of Health of the Czech Republic (grants AZV NU21-07-00247 and AZV NU21-03-00506), and Charles University Research Fund (Cooperation 43-Surgical disciplines)
DACHS: This work was supported by the German Research Council (BR 1704/6-1, BR 1704/6-3, BR 1704/6-4, CH 117/1-1, HO 5117/2-1, HE 5998/2-1, KL 2354/3-1, RO 2270/8-1 and BR 1704/17-1), the Interdisciplinary Research Program of the National Center for Tumor Diseases (NCT), Germany, and the German Federal Ministry of Education and Research (01KH0404, 01ER0814, 01ER0815, 01ER1505A and 01ER1505B).
DALS: National Institutes of Health (R01 CA048998 to M. L. Slattery).
EDRN: This work is funded and supported by the NCI, EDRN Grant (U01-CA152753).
EPIC: The coordination of EPIC is financially supported by International Agency for Research on Cancer (IARC) and also by the Department of Epidemiology and Biostatistics, School of Public Health, Imperial College London which has additional infrastructure support provided by the NIHR Imperial Biomedical Research Centre (BRC). The national cohorts are supported by: Danish Cancer Society (Denmark); Ligue Contre le Cancer, Institut Gustave Roussy, Mutuelle Générale de l'Education Nationale, Institut National de la Santé et de la Recherche Médicale (INSERM) (France); German Cancer Aid, German Cancer Research Center (DKFZ), German Institute of Human Nutrition Potsdam-Rehbruecke (DIfE), Federal Ministry of Education and Research (BMBF) (Germany); Associazione Italiana per la Ricerca sul Cancro-AIRC-Italy, Compagnia di SanPaolo and National Research Council (Italy); Dutch Ministry of Public Health, Welfare and Sports (VWS), Netherlands Cancer Registry (NKR), LK Research Funds, Dutch Prevention Funds, Dutch ZON (Zorg Onderzoek Nederland), World Cancer

Research Fund (WCRF), Statistics Netherlands (The Netherlands); Health Research Fund (FIS) - Instituto de Salud Carlos III (ISCIII), Regional Governments of Andalucía, Asturias, Basque Country, Murcia and Navarra, and the Catalan Institute of Oncology - ICO (Spain); Swedish Cancer Society, Swedish Research Council and and Region Skåne and Region Västerbotten (Sweden); Cancer Research UK (14136 to EPIC-Norfolk; C8221/A29017 to EPIC-Oxford), Medical Research Council (1000143 to EPIC-Norfolk; MR/M012190/1 to EPIC-Oxford). (United Kingdom).

EPICOLON: This work was supported by grants from Fondo de Investigación Sanitaria/FEDER (PI08/0024, PI08/1276, PS09/02368, P111/00219, PI11/00681, PI14/00173, PI14/00230, PI17/00509, 17/00878, PI20/00113, PI20/00226, Acción Transversal de Cáncer), Xunta de Galicia (PGIDIT07PXIB9101209PR), Ministerio de Economia y Competitividad (SAF07-64873, SAF 2010-19273, SAF2014-54453R), Fundación Científica de la Asociación Española contra el Cáncer (GCB13131592CAST), Beca Grupo de Trabajo “Oncología” AEG (Asociación Española de Gastroenterología), Fundación Privada Olga Torres, FP7 CHIBCHA Consortium, Agència de Gestió d’Ajuts Universitaris i de Recerca (AGAUR, Generalitat de Catalunya, 2014SGR135, 2014SGR255, 2017SGR21, 2017SGR653), Catalan Tumour Bank Network (Pla Director d’Oncologia, Generalitat de Catalunya), PERIS (SLT002/16/00398, Generalitat de Catalunya), CERCA Programme (Generalitat de Catalunya) and COST Action BM1206 and CA17118. CIBERehd is funded by the Instituto de Salud Carlos III.

ESTHER/VERDI. This work was supported by grants from the Baden-Württemberg Ministry of Science, Research and Arts and the German Cancer Aid.

Harvard cohorts : HPFS is supported by the National Institutes of Health (P01 CA055075, UM1 CA167552, U01 CA167552, R01 CA137178, R01 CA151993, and R35 CA197735), NHS by the National Institutes of Health (P01 CA087969, UM1 CA186107, R01 CA137178, R01 CA151993, and R35 CA197735), and PHS by the National Institutes of Health (R01 CA042182).

Hawaii Adenoma Study: NCI grants R01 CA072520.

HCES-CRC: the Hwasun Cancer Epidemiology Study–Colon and Rectum Cancer (HCES-CRC; grants from Chonnam National University Hwasun Hospital, HCRI15011-1).

Kentucky: This work was supported by the following grant support: Clinical Investigator Award from Damon Runyon Cancer Research Foundation (CI-8); NCI R01CA136726.

LCCS: The Leeds Colorectal Cancer Study was funded by the Food Standards Agency and Cancer Research UK Programme Award (C588/A19167).

MCCS cohort recruitment was funded by VicHealth and Cancer Council Victoria. The MCCS was further supported by Australian NHMRC grants 509348, 209057, 251553 and 504711 and by infrastructure provided by Cancer Council Victoria. Cases and their vital status were ascertained through the Victorian Cancer Registry (VCR) and the Australian Institute of Health and Welfare (AIHW), including the National Death Index and the Australian Cancer Database. BMLynch was supported by MCRF18005 from the Victorian Cancer Agency.

MEC: National Institutes of Health (R37 CA054281, P01 CA033619, and R01 CA063464).

MECC: This work was supported by the National Institutes of Health, U.S. Department of Health and Human Services (R01 CA081488, R01 CA197350, U19 CA148107, R01 CA242218, and a generous gift from Daniel and Maryann Fong.

MSKCC: The work at Sloan Kettering in New York was supported by the Robert and Kate Niehaus Center for Inherited Cancer Genomics and the Romeo Milio Foundation. Moffitt: This work was supported by funding from the National Institutes of Health (grant numbers R01 CA189184, P30 CA076292), Florida Department of Health Bankhead-Coley Grant 09BN-13, and the University of South Florida Oehler Foundation. Moffitt contributions were supported in part by the Total Cancer Care Initiative, Collaborative Data Services Core, and Tissue Core at the H. Lee Moffitt Cancer Center & Research Institute, a National Cancer Institute-designated Comprehensive Cancer Center (grant number P30 CA076292).

NCCCS I & II: We acknowledge funding support for this project from the National Institutes of Health, R01 CA066635 and P30 DK034987.

NFCCR: This work was supported by an Interdisciplinary Health Research Team award from the Canadian Institutes of Health Research (CRT 43821); the National Institutes of Health, U.S. Department of Health and Human Serivces (U01 CA074783); and National Cancer Institute of Canada grants (18223 and 18226). The authors wish to acknowledge the contribution of Alexandre Belisle and the genotyping team of the McGill University and Génome Québec Innovation Centre, Montréal, Canada, for genotyping the Sequenom panel in the NFCCR samples. Funding was provided to Michael O. Woods by the Canadian Cancer Society Research Institute.
NSHDS: The research was supported by Biobank Sweden through funding from the Swedish Research Council (VR 2017-00650, VR 2017-01737), the Swedish Cancer Society (CAN 2017/581), Region Västerbotten (VLL-841671, VLL-833291), Knut and Alice Wallenberg Foundation (VLL-765961), and the Lion's Cancer Research Foundation (several grants) and Insamlingsstiftelsen, both at Umeå University.
OSUMC: OCCPI funding was provided by Pelotonia and HNPCC funding was provided by the NCI (CA016058 and CA067941).
PLCO: Intramural Research Program of the Division of Cancer Epidemiology and Genetics and supported by contracts from the Division of Cancer Prevention, National Cancer Institute, NIH, DHHS. Funding was provided by National Institutes of Health (NIH), Genes, Environment and Health Initiative (GEI) Z01 CP 010200, NIH U01 HG004446, and NIH GEI U01 HG 004438.
SEARCH: The University of Cambridge has received salary support in respect of PDPP from the NHS in the East of England through the Clinical Academic Reserve. Cancer Research UK (C490/A16561); the UK National Institute for Health Research Biomedical Research Centres at the University of Cambridge.
SELECT: Research reported in this publication was supported in part by the National Cancer Institute of the National Institutes of Health under Award Numbers U10 CA037429 (CD Blanke), and UM1 CA182883 (CM Tangen/IM Thompson). The content is solely the responsibility of the authors and does not necessarily represent the official views of the National Institutes of Health.
SMS and REACHS: This work was supported by the National Cancer Institute (grant P01 CA074184 to J.D.P. and P.A.N., grants R01 CA097325, R03 CA153323, and K05 CA152715 to P.A.N., and the National Center for Advancing Translational Sciences at the National Institutes of Health (grant KL2 TR000421 to A.N.B.-H.)
The Swedish Low-risk Colorectal Cancer Study: The study was supported by grants from the Swedish research council; K2015-55X-22674-01-4, K2008-55X-20157-03-3, K2006-72X-20157-01-2 and the Stockholm County Council (ALF project).
Swedish Mammography Cohort and Cohort of Swedish Men: This work is supported by the Swedish Research Council /Infrastructure grant, the Swedish Cancer Foundation, and the Karolinska Institute´s Distinguished Professor Award to Alicja Wolk.
UK Biobank: This research has been conducted using the UK Biobank Resource under Application Number 8614
VITAL: National Institutes of Health (K05 CA154337).
The WHI program is funded by the National Heart, Lung, and Blood Institute, National Institutes of Health, U.S. Department of Health and Human Services through contracts 75N92021D00001, 75N92021D00002, 75N92021D00003, 75N92021D00004, 75N92021D00005

Acknowledgements:

ASTERISK: We are very grateful to those, without whom this project would not have existed. We also thank all those who agreed to participate in this study, including the patients and the healthy control persons, as well as all the physicians, technicians and students.

CCFR: The Colon CFR graciously thanks the generous contributions of their study participants, dedication of study staff, and the financial support from the U.S. National Cancer Institute, without which this important registry would not exist. The authors would like to thank the study participants and staff of the Seattle Colon Cancer Family Registry and the Hormones and Colon Cancer study (CORE Studies).

CLUE II: We thank the participants of Clue II and appreciate the continued efforts of the staff at the Johns Hopkins George W. Comstock Center for Public Health Research and Prevention in the conduct of the Clue II Cohort Study. Cancer data was provided by the Maryland Cancer Registry, Center for Cancer Prevention and Control, Maryland Department of Health, with funding from the State of Maryland and the Maryland Cigarette Restitution Fund. The collection and availability of cancer registry data is also supported by the Cooperative Agreement NU58DP006333, funded by the Centers for Disease Control and Prevention. Its contents are solely the responsibility of the authors and do not necessarily represent the official views of the Centers for Disease Control and Prevention or the Department of Health and Human Services.

COLON and NQplus: the authors would like to thank the COLON and NQplus investigators at Wageningen University & Research and the involved clinicians in the participating hospitals.

CORSA: We kindly thank all individuals who agreed to participate in the CORSA study. Furthermore, we thank all cooperating physicians and students and the Biobank Graz of the Medical University of Graz.

CPS-II : The authors express sincere appreciation to all Cancer Prevention Study-II participants, and to each member of the study and biospecimen management group. The authors would like to acknowledge the contribution to this study from central cancer registries supported through the Centers for Disease Control and Prevention's National Program of Cancer Registries and cancer registries supported by the National Cancer Institute's Surveillance Epidemiology and End Results Program. The authors assume full responsibility for all analyses and interpretation of results. The views expressed here are those of the authors and do not necessarily represent the American Cancer Society or the American Cancer Society – Cancer Action Network.

Czech Republic CCS: We are thankful to all clinicians in major hospitals in the Czech Republic, without whom the study would not be practicable. We are also sincerely grateful to all patients participating in this study.

DACHS: We thank all participants and cooperating clinicians, and everyone who provided excellent technical assistance.

EDRN: We acknowledge all contributors to the development of the resource at University of Pittsburgh School of Medicine, Department of Gastroenterology, Department of Pathology, Hepatology and Nutrition and Biomedical Informatics.

EPIC: Where authors are identified as personnel of the International Agency for Research on Cancer/World Health Organization, the authors alone are responsible for the views expressed in this article and they do not necessarily represent the decisions, policy or views of the International Agency for Research on Cancer/World Health Organization.

EPICOLON: We are sincerely grateful to all patients participating in this study who were recruited as part of the EPICOLON project. We acknowledge the Spanish National DNA Bank, Biobank of Hospital Clínic–IDIBAPS and Biobanco Vasco for the availability of the samples. The work was carried out (in part) at the Esther Koplowitz Centre, Barcelona.

Harvard cohorts: The study protocol was approved by the institutional review boards of the Brigham and Women’s Hospital and Harvard T.H. Chan School of Public Health, and those of participating registries as required. We acknowledge Channing Division of Network Medicine, Department of Medicine, Brigham and Women's Hospital as home of the NHS. The authors would like to acknowledge the contribution to this study from central cancer registries supported through the Centers for Disease Control and Prevention’s National Program of Cancer Registries (NPCR) and/or the National Cancer Institute’s Surveillance, Epidemiology, and End Results (SEER) Program. Central registries may also be supported by state agencies, universities, and cancer centers. Participating central cancer registries include the following: Alabama, Alaska, Arizona, Arkansas, California, Colorado, Connecticut, Delaware, Florida, Georgia,

Hawaii, Idaho, Indiana, Iowa, Kentucky, Louisiana, Massachusetts, Maine, Maryland, Michigan, Mississippi, Montana, Nebraska, Nevada, New Hampshire, New Jersey, New Mexico, New York, North Carolina, North Dakota, Ohio, Oklahoma, Oregon, Pennsylvania, Puerto Rico, Rhode Island, Seattle SEER Registry, South Carolina, Tennessee, Texas, Utah, Virginia, West Virginia, Wyoming. The authors assume full responsibility for analyses and interpretation of these data.

Kentucky: We would like to acknowledge the staff at the Kentucky Cancer Registry.

LCCS: We acknowledge the contributions of Jennifer Barrett, Robin Waxman, Gillian Smith and Emma Northwood in conducting this study.

NCCCS I & II: We would like to thank the study participants, and the NC Colorectal Cancer Study staff.

NSHDS investigators thank the Västerbotten Intervention Programme, the Northern Sweden MONICA study, the Biobank Research Unit at Umeå University and Biobanken Norr at Region Västerbotten for providing data and samples and acknowledge the contribution from Biobank Sweden, supported by the Swedish Research Council.

PLCO: The authors thank the PLCO Cancer Screening Trial screening center investigators and the staff from Information Management Services Inc and Westat Inc. Most importantly, we thank the study participants for their contributions that made this study possible.

Cancer incidence data have been provided by the District of Columbia Cancer Registry, Georgia Cancer Registry, Hawaii Cancer Registry, Minnesota Cancer Surveillance System, Missouri Cancer Registry, Nevada Central Cancer Registry, Pennsylvania Cancer Registry, Texas Cancer Registry, Virginia Cancer Registry, and Wisconsin Cancer Reporting System. All are supported in part by funds from the Center for Disease Control and Prevention, National Program for Central Registries, local states or by the National Cancer Institute, Surveillance, Epidemiology, and End Results program. The results reported here and the conclusions derived are the sole responsibility of the authors.

SEARCH: We thank the SEARCH team

SELECT: We thank the research and clinical staff at the sites that participated on SELECT study, without whom the trial would not have been successful. We are also grateful to the 35,533 dedicated men who participated in SELECT.

WHI: The authors thank the WHI investigators and staff for their dedication, and the study participants for making the program possible. A full listing of WHI investigators can be found at: http://www.whi.org/researchers/Documents%20%20Write%20a%20Paper/WHI%20Investigator%20Short%20List.pdf
